# Supplementary material for: Core Transcription Factors Promote Induction of PAX3-Positive Skeletal Muscle Stem Cells
Source: Stem Cell Reports. 2019 Jul 25;13(2):352–65. doi: 10.1016/j.stemcr.2019.06.006 (PMC6700474; doi:10.1016/j.stemcr.2019.06.006)
Supplement: Document S1. Figures S1–S5 and Tables S2 and S3 [file mmc1.pdf]

Stem Cell Reports, Volume *13*

Supplemental Information

Core Transcription Factors Promote Induction of PAX3-Positive Skeletal Muscle Stem Cells

Takahiko Sato, Koki Higashioka, Hidetoshi Sakurai, Takuya Yamamoto, Naoki Goshima, Morio Ueno, and Chie Sotozono

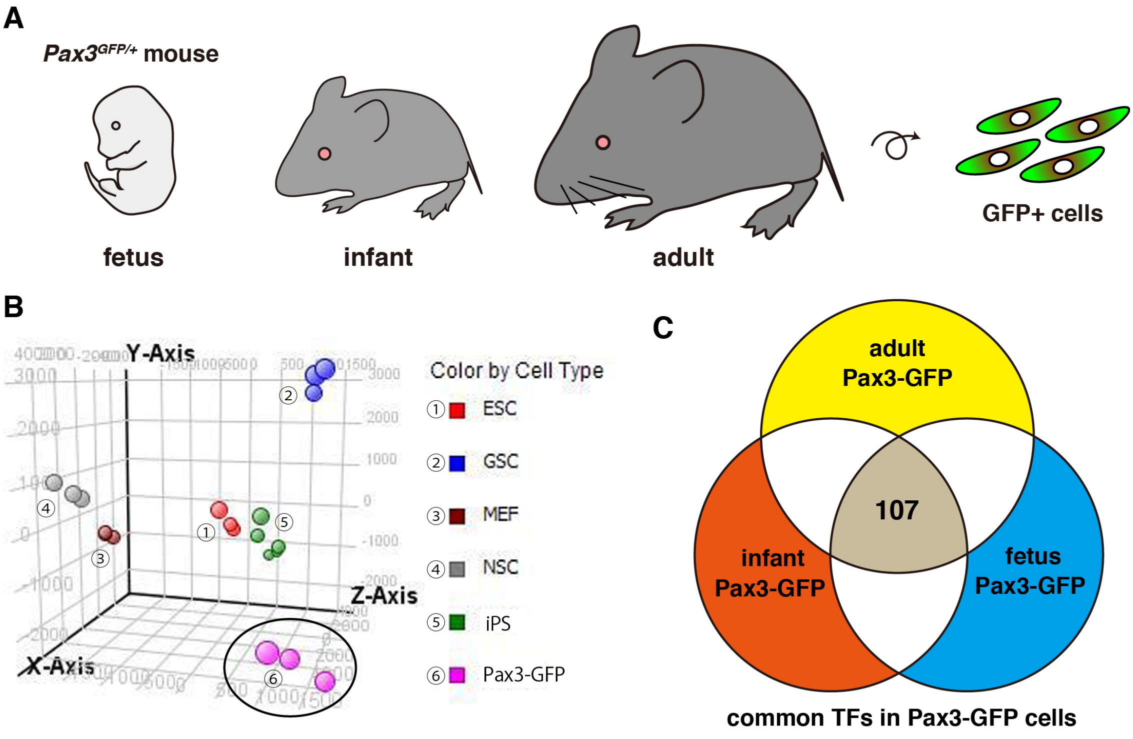

Figure S1. Gene profile from myogenic precursors and adult muscle stem cells of Pax3$^{GFP/+}$ mice.

(A) 3 different developmental stages (fetus, infant, and adult mouse) were used for isolating Pax3-GFP expressing cells of skeletal muscle tissue.

(B) Principal component analysis (PCA) with Pax3-GFP cells and other types of cells including ESC (mouse embryonic stem cells), GSC (mouse germline stem cells), MEF (mouse embryonic fibroblasts), NSC (mouse neural stem cells), and iPS (mouse induced pluripotent stem cells).

(C) 107 selected genes, which are highly expressed in Pax3-expressing cells derived from 3 different stages of *Pax3$^{GFP/+}$* mice, relative to other cells (see Table S1).

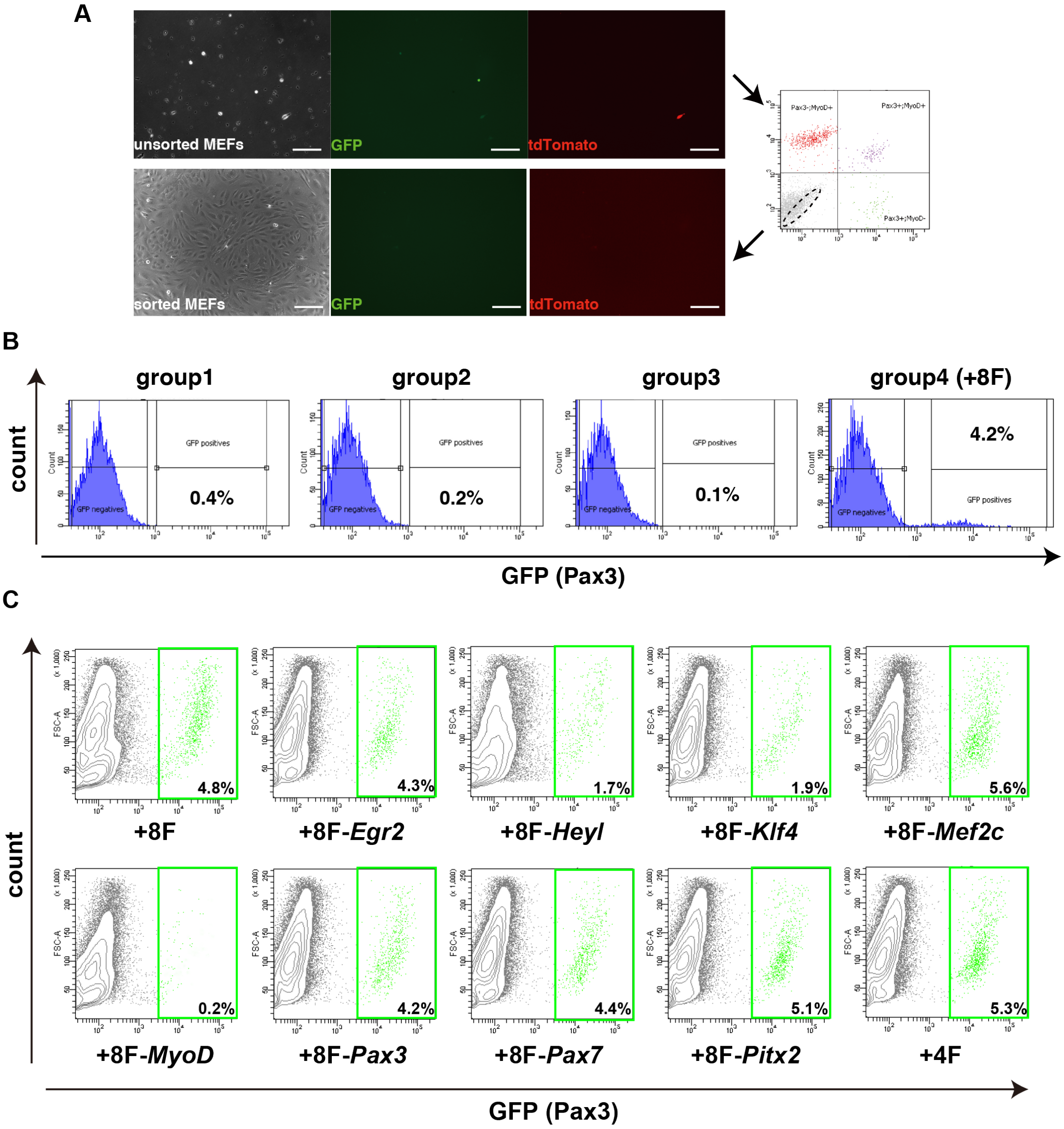

Figure S2. FACS analyses with Pax3$^{GFP/+}$ MEFs for reprogramming. (A) Fluorescent signals of GFP (Green) or tdTomato (Red) could be detected in mouse embryonic fibroblasts (MEFs) isolated from *Pax3-GFP; MyoD-tdTomato* embryos (upper panels). Sorted MEFs had no signal of any fluorescent proteins (lower panels). Scale bars; 50 μm. (B) FACS data of Pax3-GFP cells expressing selected candidates, named as Group1 (*Dmrt2, Foxp2, Hey1, Lbx1, Pbx1, Mef2d, Myf5, Six1, Tshz1, Tshz3*), Group2 (*Myog, Nfib, Pknox2, Prox1, Pere, Rora, Runx1, Sox6, Tbx1, Tbx15*), Group3 (*Tbx4, Tef, Tfdp2, Thra, Yy1, Zeb2, Zfp445, Zhx1, Zkscan17, Zkscan3*), Group4 (*Egr2, Heyl, Klf4, Mef2c, MyoD, Pax3, Pax7, Pitx2*) were used for infected into MEFs. (C) Narrowing down from 8 candidate transcription factors for PAX3-expressing myogenic cells derived from sorted MEFs.

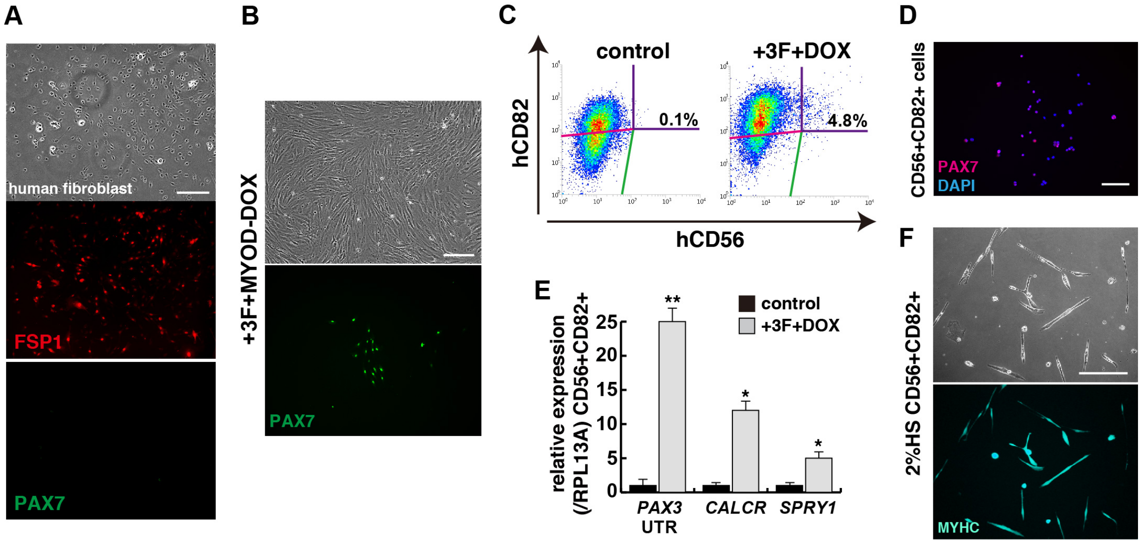

Figure S3. **4 transcription factors convert human fibroblasts into myogenic stem cells.** (A) Immunostaining of anti-FSP1 (labelled with Alexa594, red) and anti-PAX7 (labelled with Alexa488, green) antibodies with human dermal fibroblasts. (B) PAX3, HEYL, KLF4 (+3F) and transient DOX-treatment for 72 hours (+MYOD-DOX) induced PAX7-expressing cells from human dermal fibroblasts (labelled with Alexa488, green). (C) FACS profile of CD56 and CD82 cells from cultured human dermal fibroblasts with or without PAX3, HEYL, KLF4 and DOX treatment for 72 hours (control or +3F+DOX/control). (D) Immunostaining with anti-PAX7 antibody with CD56+CD82+ double positive sorted cells. PAX7; red, DAPI; blue. (E) Transcriptome analyses of induced CD56+CD82+ cells with or without the 4 factors. All error bars indicate ±SEM (n=3). *P*-values are determined by *t*-test from a two-tailed distribution. (F) MYHC (light blue) was stained with myogenic differentiated cells with 2% of horse serum (2%HS) from CD56+CD82+ sorted cells for 7 days. All scale bars; 50 μm.

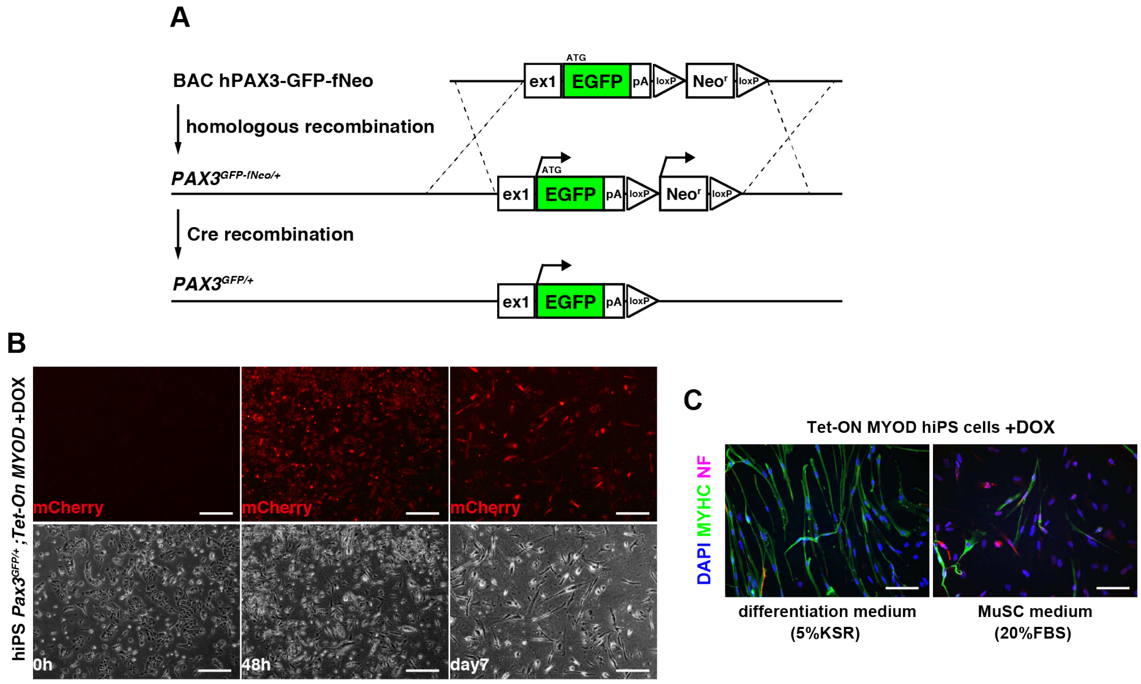

Figure S4. Strategy of GFP-targeting into the PAX3 knock-in and Tet-ON MYOD1 human iPS cells. (A) Schematic diagram of the human PAX3 locus and targeting construct. The BAC DNA construct contains more than 100kb of PAX3 genomic sequence. An EGFP reporter gene followed by floxed neomycin (fNeo) selection marker replaces the coding sequence in exon 1 of PAX3 (BAC *hPAX3-GFP-fNeo*). After homologous recombination in 201B7 hiPS cells, cells were selected by the administration of G418, and the knockin allele was confirmed by qPCR with genomic DNA and sequencing reaction ($PAX3^{GFP\text{-}fNeo/+}$). The fNeo cassette is removed with expression of CAG-Cre plasmid. This generates the $PAX3^{GFP/+}$ allele in hiPS cells. (B) $Pax3^{GFP/+}$*; Tet-On MYOD* hiPS cells were differentiated into myogenic cells with the administration of DOX for 7 days. (C) Immunostaining of terminally differentiated myotubes derived from hiPS cells with the administration of DOX for a week and 5% of Knockout Serum Replacement (KSR) was carried out according to an optimized protocol. (Tanaka et al. 2013, left panel). Neural cells appear in cultured cells with 20% of FBS instead of KSR (red, right panel). DAPI (blue), Myosin Heavy Chain (MyHC; labelled with Alexa488, Green), Neurofilament (NF; labelled with Alexa647, Red). All scale bar; 50 µm.

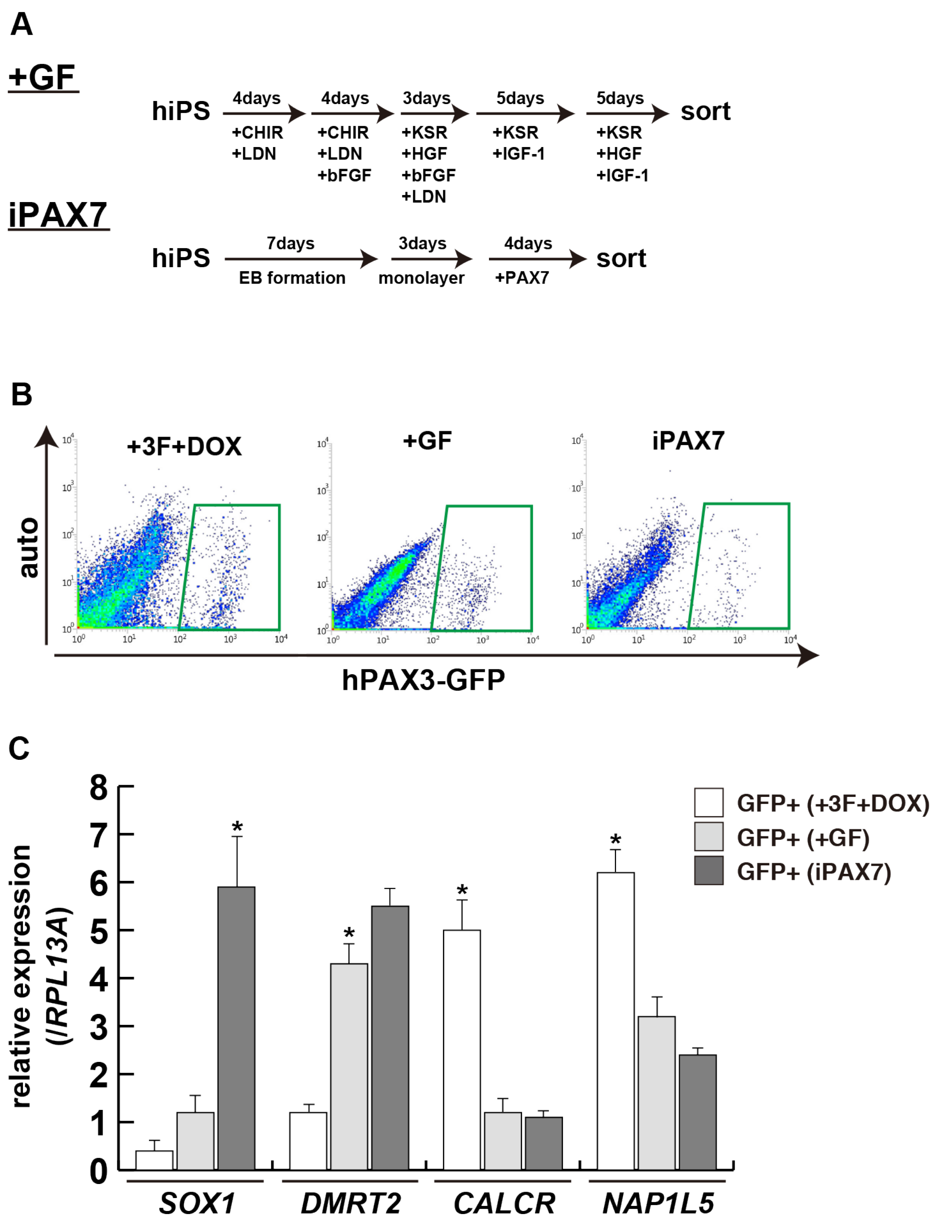

Figure S5. The comparison of induction methods for PAX3-expressing muscle stem cells from human iPS cells. (A) Reported schematic differentiation protocols for isolating PAX3-GFP myogenic cells derived from hiPS cells (iPAX7, Darabi et al. 2012.; +GF, Chal et al. 2015). (B) FACS profile of induced PAX3-GFP cells from hiPS cells. (C) Transcriptional analyses with PAX3-GFP positive cells induced by transient DOX treatment plus 3 transcription factors (+3F+DOX), +growth factors (+GF), and transient PAX7 (+iPAX7). Ectodermal and Mesodermal GFP-expressing cells, marked by *SOX1* (neural) and *DMRT2* (dermomyotome); *CALCL* and *NAP1L5* were used as markers for adult muscle satellite cells. All error bars indicate ±SEM (n=3). *P*-values are determined by Dunnett's multiple-comparisons test. *P<0.01.

Table S2. **Summarized profile of Table S1**.

38 candidate genes which show high expression in Pax3-GFP muscle stem cells.

MuSC_E16	MuSC_1wk	MuSC_12wk	ESC	MEF	GSC	NSC	Gene Symbol
234.9	185.0	113.6	4.8	4.8	4.7	4.7	Dmrt2
2286.2	5709.4	13794.6	10.1	161.7	237.7	93.0	Egr2
263.4	278.7	278.0	8.4	118.4	7.7	13.7	Foxp2
466.4	674.9	892.0	100.2	23.8	252.3	78.9	Hey1
189.4	790.0	749.6	7.5	7.3	7.4	7.2	Heyl
536.2	415.1	1637.4	224.4	262.6	93.6	10.4	Klf4
140.2	86.1	74.6	5.0	5.1	4.9	4.9	Lbx1
765.7	420.5	509.4	9.5	178.2	8.5	184.5	Pbx1
326.3	725.9	1909.8	6.3	22.7	8.8	188.5	Mef2c
385.8	521.1	381.8	16.4	109.3	76.7	149.0	Mef2d
5075.5	4503.2	9603.6	10.1	10.2	8.6	8.8	Myf5
960.1	2266.2	3791.0	3.5	3.5	3.4	3.4	Myod1
10523.2	12490.2	4666.3	6.1	6.1	5.8	5.8	Myog
6651.9	7772.7	5271.2	23.5	425.0	77.3	2492.1	Nfib
231.6	1174.9	1542.6	17.9	20.7	15.9	171.2	Pax3
3043.4	3078.9	4765.1	4.6	4.6	4.5	4.4	Pax7
1936.7	2850.5	1779.0	92.1	164.1	9.9	9.6	Pitx2
321.8	758.6	224.2	6.6	9.2	5.4	43.2	Pknox2
161.6	733.3	473.6	7.6	7.0	6.7	14.0	Prox1
804.1	1129.9	864.9	162.5	144.2	367.6	392.3	Rere
319.0	543.5	2214.3	41.7	24.8	45.9	41.8	Rora
125.3	217.6	1614.6	6.2	28.0	8.7	5.6	Runx1
429.6	399.2	301.6	27.3	80.0	220.9	11.3	Six1
406.8	481.9	281.1	14.4	17.6	13.5	153.4	Sox6
182.4	228.1	104.4	5.9	7.9	7.1	5.9	Tbx1
306.4	302.1	258.5	6.6	248.6	9.2	64.5	Tbx15
76.0	70.6	127.8	5.5	16.5	5.2	5.3	Tbx4
816.2	850.9	657.6	194.7	229.1	438.6	315.9	Tef
2527.8	2323.1	1054.0	610.8	128.8	198.6	265.8	Tfdp2
242.2	702.1	447.1	5.8	34.3	16.7	222.7	Thra
2499.5	2727.7	1730.1	7.1	452.2	15.8	466.1	Tshz1
686.8	740.7	934.0	16.5	129.1	16.6	86.2	Tshz3
1336.7	995.4	1132.6	352.3	472.0	61.7	594.6	Yy1
854.9	4935.4	3900.5	11.5	249.2	8.0	147.3	Zeb2
441.2	887.0	525.6	165.1	141.6	177.6	273.0	Zfp445
596.6	1129.1	743.8	145.4	270.0	128.9	364.6	Zhx1
679.4	871.6	733.0	374.6	156.2	49.4	463.5	Zkscan17
1039.6	755.8	678.5	77.9	164.8	157.7	308.7	Zkscan3

Table S3. Primers for the expression analysis by RT-qPCR of the mRNAs indicated.

for mouse

Gene	Sequence	Target position	Product
Rpl13a	5’-GTGGTCCCTGCTGCTCTCAAG-3’	exon6	151bp
	5’-CGATAGTGCATCTTGGCCTTTT-3’	exon7	
Pax7 (UTR)	5’-CCCTTCCTACAGGAAACCCTCT-3’	exon10	72bp
	5’-CTGAACCAGACCTGGACGCG-3’	exon10	
MyoD (UTR)	5’-GCGCTCTTCCTTTCCTCATAG-3’	exon2	90bp
	5’-GGGCTCCAGAAAGTGACAAAC-3’	exon3	
Myog	5’-CAACCAGGAGGAGCGCGATCTCCG-3’	exon1	93bp
	5’-GGCGCTGTGGGAGTTGCATTCACT-3’	exon2	
Calcr	5’-ATCTGGTGCGGCGGGAT-3’	exon10	94bp
	5’-CCCTCGCAGAGCATCCAGAA-3’	exon12	
Spry1	5’-ATGGATTCCCCAAGTCAGCAT-3’	exon3	93bp
	5’-CCTGTCATAGTCTAACCTCTGCC-3’	exon3	
Sdc4	5’-GTCCCCGGAGAGTCGATTC-3’	exon1	193bp
	5’-GCACCAAGGGCTCAATCACTT-3’	exon3	
Myh3	5’-TCCAAACCGTCTCTGCACTGTT-3’	exon17	84bp
	5’-AGCGTACAAAGTGTGGGTGTGT-3’	exon18	
Myh7	5’-ATGCTGACAGATCGGGAGAA -3’	exon	181bp
	5’-GGTTGGCTTGGATGATTTGA-3’	exon	
Myh1	5’-TCTGCAGACGGAGTCAGGT-3’	exon33	94bp
	5’-TTGAGTGAATGCCTGTTTGC-3’	exon34	
Sox1	5’-CCTCGGATCTCTGGTCAAGT-3’	exon1	107bp
	5’-GCAGGTACATGCTGATCATCTC-3’	exon1	
Sox10	5’-ATGTCAGATGGGAACCCAGA-3’	exon2	74bp
	5’-GTCTTTGGGGTGGTTGGAG-3’	exon3	

for human

RPL13A	5’-CCCTGGAGGAGAAGAGGAAA-3’	exon11	91bp
	5’-ACGTTCTTCTCGGCCTGTTT-3’	exon12	
PAX3	5’-AGGAAGGAGGCAGAGGAAAG-3’	exon6	174bp
	5’-CAGCTGTTCTGCTGTGAAGG-3’	exon10	
PAX3(UTR)	5’-AAGCCAGCTGACTGTTCCAG-3’	exon12	74bp
	5’-CCGCAAGATGTTGTTGACAT-3’	exon12	
PAX7	5’-GGGATTCCCTTTGGAAGTGT-3’	exon1-2	198bp
	5’-CGGCAAAGAATCTTGGAGAC-3’	exon2	
CALCR	5’-CCCTTTGCTTCTATTGAGCTG-3’	exon2	69bp
	5’-GGTAATAGCATGGATAGTGGTTGGt-3’	exon3	
MYF5	5’-CTATAGCCTGCCGGGACAGA-3’	exon1	95bp
	5’-TGGACCAGACAGGACTGTTACAT-3’	exon3	
DMRT2	5’-GAACCACCAAGCAAGGACTTC-3’	exon5	75bp
	5’-CCCAGACCCTGAATACTGCAT-3’	exon5	
NFIX	5’-TGACTCCTCCATCACCTTCA-3’	exon8	71bp
	5’-GGGTCCGATGCTGACAAA-3’	exon9	
NAP1L5	5’-GTGTGCATGGACCTTGGAG-3’	exon1	62bp
	5’-CCTCCTCGTCATCCTCGTACT-3’	exon1	
SPRY1	5’-TCCCTGGTCATAGGTCTGAAAG-3’	exon4	187bp
	5’-TGCCGGTTACAGGCCAAAC-3’	exon4	
MSGN1	5’-GGAGAAGCTCAGGATGAGGA-3’	exon1	145bp
	5’-GTCTGTGAGTTCCCCGATGT-3’	exon1	
TBX6	5’-GAACGGCAGAAACTGTAAGAGG-3’	exon5	101bp
	5’-GTGTGTCTCCGCTCCCATAG-3’	exon6	
PDGFRa	5’-AACCGTGTATAAGTCAGGGGA-3’	exon4	126bp
	5’-ATTTCTTCCAGCATTGTGAT-3’	exon6	
SOX1	5’-ACCAGGCCATGGATGAAG-3’	exon1	67bp
	5’-CTTAATTGCTGGGGAATTGG-3’	exon1	
SOX10	5’-CGGACCAGTACCCGCACCT-3’	exon2	87bp
	5’-GGCGCTTGTCACTTTCGTTCA-3’	exon3	
