# Supplementary material for: Core Transcription Factors Promote Induction of PAX3-Positive Skeletal Muscle Stem Cells
Source: Stem Cell Reports. 2019 Jul 25;13(2):352–65. doi: 10.1016/j.stemcr.2019.06.006 (PMC6700474; doi:10.1016/j.stemcr.2019.06.006)
Supplement: Document S2. Article plus Supplemental Information [file mmc3.pdf]

Stem Cell Reports
Article
OPEN ACCESS

Core Transcription Factors Promote Induction of PAX3-Positive Skeletal Muscle Stem Cells

Takahiko Sato,[1,6,7,*] Koki Higashioka,[1,2] Hidetoshi Sakurai,[3] Takuya Yamamoto,[4,7] Naoki Goshima,[5] Morio Ueno,[1] and Chie Sotozono[1]
[1]Department of Ophthalmology, Kyoto Prefectural University of Medicine, Kyoto, Japan
[2]Department of Biomedical Engineering, Faculty of Life and Medical Sciences, Doshisha University, Kyotanabe, Japan
[3]Department of Clinical Application, Center for iPS Cell Research and Application, Kyoto University, Kyoto, Japan
[4]Department of Life Science Frontiers, Center for iPS Cell Research and Application, Kyoto University, Kyoto, Japan
[5]Molecular Profiling Research Center for Drug Discovery, National Institute of Advanced Industrial Science and Technology, Tokyo, Japan
[6]Department of Anatomy, Fujita Health University, Toyoake, Aichi, Japan
[7]AMED-CREST, AMED, 1-7-1 Otemachi, Chiyoda, Tokyo, Japan
*Correspondence: takahiko@koto.kpu-m.ac.jp
https://doi.org/10.1016/j.stemcr.2019.06.006

SUMMARY

The use of adult skeletal muscle stem cells (MuSCs) for cell therapy has been attempted for decades, but still encounters considerable difficulties. MuSCs derived from human induced pluripotent stem cells (hiPSCs) are promising candidates for stem cell therapy to treat Duchenne muscular dystrophy (DMD). Here we report that four transcription factors, HEYL, KLF4, MYOD, and PAX3, selected by comprehensive screening of different MuSC populations, enhance the derivation of PAX3-positive myogenic progenitors from fibroblasts and hiPSCs, using medium that promotes the formation of presomitic mesoderm. These induced PAX3-positive cells contribute efficiently to the repair of DMD-damaged myofibers and also reconstitute the MuSC population. These studies demonstrate how a combination of core transcription factors can fine-tune the derivation of MuSCs capable of contributing to the repair of adult skeletal muscle.

INTRODUCTION

The muscular dystrophies are a group of inherited skeletal muscle disorders characterized clinically by progressive muscle weakness and wasting. Duchenne muscular dystrophy (DMD) is among the most common and severe forms of these muscle diseases. DMD is caused by mutations or deletions in the *Dmd* gene, which lead to loss of muscle fiber integrity and continuous muscle damage. This damage leads to the rapid wasting of skeletal muscles, and there is as yet no cure, although a number of promising approaches are being developed to retard the progression of DMD symptoms (Guiraud et al., 2015). Cell replacement therapy uses extrinsic myogenic cells that express functional dystrophin protein to replace the abnormal skeletal muscle tissue of individuals with DMD (Negroni et al., 2016).

Skeletal muscle tissue has its own intrinsic repair and maintenance system, which depends on adult muscle stem cells (MuSCs). MuSCs are closely associated with the muscle fiber, hence their description as satellite cells, and are normally quiescent, but begin to proliferate in response to muscle injury or during intense exercise. They enter the myogenic differentiation program, fuse with damaged myofibers or form *de novo* fibers, and also reconstitute the quiescent MuSC population (Collins et al., 2005). Mouse MuSCs that have no *Dmd* mutation, when engrafted into the damaged muscle of DMD mice, contribute to the regeneration of DMD myofibers, which are now positive for functional dystrophin protein (Cerletti et al., 2008).

Skeletal muscle regeneration is regulated by families of transcription factors also essential for skeletal muscle formation in the embryo. PAX3, a paired box transcription factor, is expressed in the paraxial mesoderm of newly forming somites and then in the dorsal compartment of somites, the dermomyotome, which will give rise to myogenic progenitor cells. *Pax3*, and its sister gene, *Pax7*, also expressed later in all muscle progenitor cells, lie upstream of the MYOD family of basic-helix-loop-helix transcription factors that play an essential role in muscle formation. The MYOD family is specific to skeletal muscle, whereas PAX3 and PAX7 are also expressed in some cells of the central nervous system, and PAX3 plays an important role in the development of neural crest cells (Buckingham and Relaix, 2015). In the skeletal muscle context, the population of PAX3/PAX7-positive progenitors is essential for myogenic growth during development and is also regarded as the source of adult MuSCs (Gros et al., 2005). Recent studies have revealed that core transcription factors, expressed in adult stem cells of a number of different tissues, can induce and maintain a specific cell lineage directly when introduced into pluripotent stem cells (PSCs) and into other cell types (Morris, 2016). A classic example of such tissue-specific conversion is provided by the MYOD family, which when expressed in non-muscle cells activates the endogenous *MyoD* family genes and leads to the establishment of the gene regulatory network required for the subsequent formation of skeletal muscle (Weintraub et al., 1989).

 © 2019 The Author(s).
This is an open access article under the CC BY license (http://creativecommons.org/licenses/by/4.0/).

In the study reported here, we established a defined system to induce myogenic stem cells from fibroblasts, identified by expression of a Pax3-GFP reporter. This is based on a combination of transcription factors which, together with appropriate mesodermal culture medium, are more effective than any single factor in generating PAX3-positive myogenic progenitors that when engrafted into dystrophic mouse muscle efficiently regenerate dystrophin-positive fibers and reconstitute the PAX7-positive stem cell compartment. These results provide new insight into the core transcription factors that underlie the conversion of non-muscle cells to myogenic progenitors of potential therapeutic interest.

RESULTS

MYOD Induces Myogenic Cell Conversion, but Not PAX3-Positive Muscle Stem Cells

We have developed *Pax3*$^{GFP/+}$;*MyoD-Cre*; *Rosa26*$^{CAG-LSL-tdTomato/+}$ (*Pax3-GFP*;*MyoD-tdTomato*) compound mice to visualize GFP-positive MuSCs (green in Figure 1A) and MYOD-primed myogenic cells (red in Figure 1A). Mouse embryonic fibroblasts isolated from this mouse line (Figures S1 and S2A) were transfected with a *Pax3*- and/or *MyoD*-expression pMX vector. *MyoD*- or *Pax3*+*MyoD*-infected cells are gradually converted into myogenic cells, which express the endogenous *MyoD* gene, shown as tdTomato-positive cells (Figure 1B), but not Pax3-GFP-positive cells corresponding to myogenic progenitor/muscle stem cells (right panels in Figure 1B). We could detect MyoD-primed, tdTomato-positive cells from day 3 after *MyoD* transfection, and the proportion of these tdTomato-positive cells increased for 2 weeks after the transfection, whereas Pax3-GFP-positive cells were rarely detected (Figure 1C). Expression of *Pax3* and *MyoD* was not enough to induce PAX3-positive cells from fibroblasts.

Transcription Factors that Induce Pax3-Positive Myogenic Cells

Next, we investigated the expression of potential regulatory genes in Pax3-GFP-positive cells isolated from skeletal muscle of fetal, postnatal, and adult mice (Figure S1A). We used these three sources of cells to identify genes that are common to the MuSC identity, irrespective of other features of the cells at different developmental stages. Genes encoding transcription factors that are highly expressed in all three conditions, compared with other cell types, were selected (Figures S1B and S1C; Tables S1 and S2). We first investigated whether expression of a combination of these transcription factors could induce Pax3-GFP-positive cells derived from mouse embryonic fibroblasts of *Pax3-GFP*;*MyoD-tdTomato* embryos. Pax3-GFP-expressing cells were generated most efficiently by a mixture of eight transcription factors (+8F), *Egr2*, *Heyl*, *Klf4*, *Mef2c*, *MyoD*, *Pax3*, *Pax7*, and *Pitx2*, which were highly expressed in all three PAX3-positive muscle progenitor/stem cell populations (Figures 2A, 2B, and S3B). We attempted to determine which of the eight transcription factors were essential for the induction of PAX3-positive MuSCs. *Pax3*, *Heyl*, *Klf4*, and *MyoD* had the ability to induce Pax3-GFP cells effectively (Figure S2C). However, MYOD protein could not be detected in adult quiescent MuSCs as previously reported (Montarras et al., 2005). We therefore controlled the expression level of MYOD with doxycycline (DOX). *Pax3*, *Heyl*, *Klf4* (3F), and constant MYOD (++DOX) could also induce Pax3-GFP as seen for 8F; however, 3F and transient MYOD expression for 72 h (+DOX) effectively enhanced the population of GFP-expressing cells (Figure 2C). These induced GFP-positive cells, selected by fluorescence-activated cell sorting (FACS), were detected as Pax7-positive cells (Figure 2D). These GFP-positive cells also expressed endogenous *Pax7*, *Calcitonin receptor*, *Sprouty1*, and *Syndecan4* transcripts, markers of muscle satellite cells (Fukada et al., 2007; Shea et al., 2010), and did not show elevated levels of transcripts for *Sox1* or *Sox10*, markers of neural or neural crest cells (Figure 2E). To compare induced GFP-positive cells with endogenous Pax3-GFP MuSCs, we expanded both populations on cell-culture dishes for 4 days. The numbers of growing cells were similar, although induced GFP-positive cells had a tendency to form aggregates (lower panel in Figure 2F) and less tendency to spontaneously form muscle fibers (Figure 2F), with maintenance of mononucleated progenitors reflected by a higher level of *Pax7* transcripts (Figure 2G). Both GFP-positive cell populations underwent myogenic differentiation when cultured under low serum conditions, as shown by staining for skeletal muscle myosin heavy chain (MyHC, Figure 2H). Differentiated MyHC-positive muscle cells from induced GFP cells were elongated and formed fewer, thinner fibers (Figure 2I). In these cultures, compared with differentiated muscle satellite cells, transcripts of *Pax7* were higher indicating more maintenance of muscle progenitors, with similar levels of transcripts for embryonic MyHC (*Myh3*) and lower levels of transcripts of *Myh7* and *Myh1* encoding slow and fast adult MyHCs, respectively (Figure 2J). These results suggest that the induced GFP cells have a less mature skeletal muscle phenotype under these culture conditions. In conclusion, the four factors (4F) have the competence to convert mouse embryonic fibroblasts to PAX3- and PAX7-positive cells that have myogenic differentiation potential as indicated by the activation of genes for skeletal muscle-specific proteins such as *Myogenin* (*Myog*) and skeletal muscle MyHC. This was also the case for human dermal fibroblasts (Figure S3).

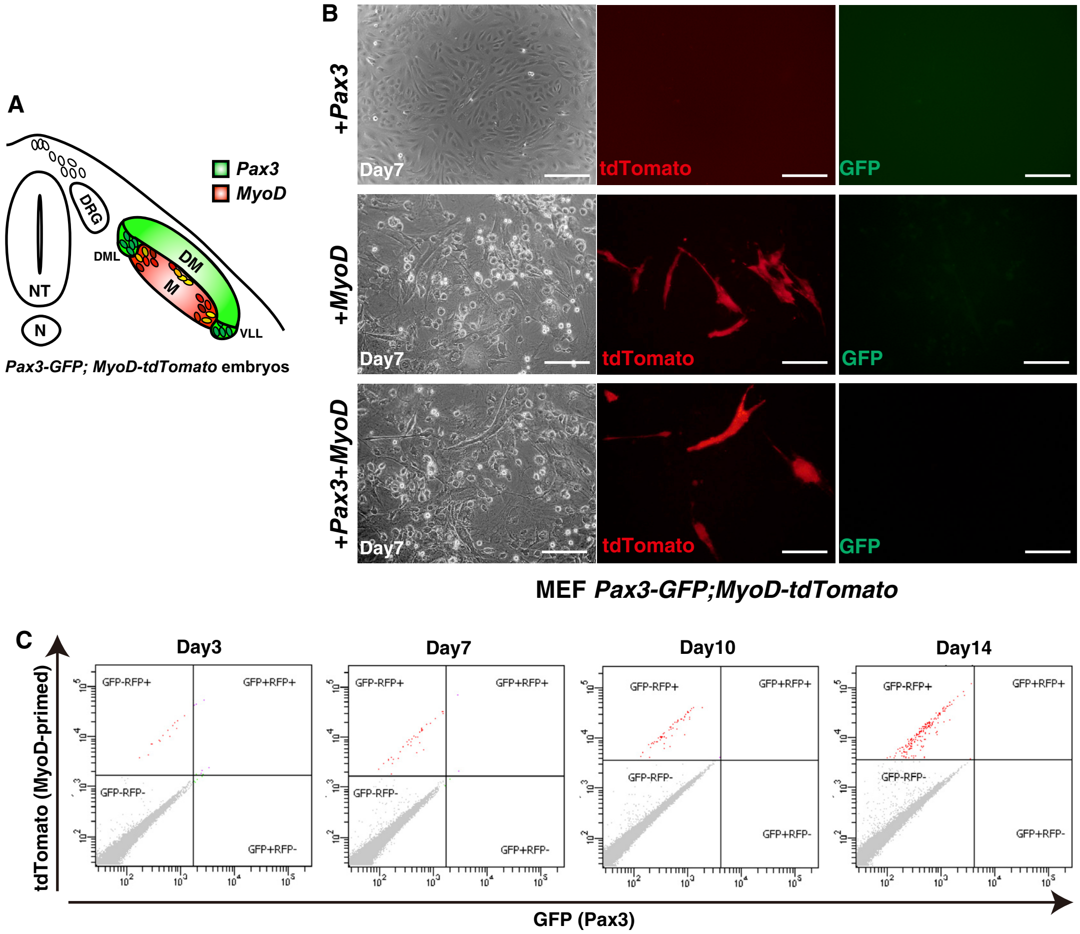

Figure 1. MYOD and PAX3 Are Not Sufficient to Induce Myogenic Stem Cells, but Result in Differentiated Cells

(A) Schematic representation of skeletal muscle development in mouse embryogenesis. *Pax3*$^{GFP/+}$ (*Pax3-GFP*) indicates dermomyotome (DM; green), and *MyoD-Cre;Rosa26*$^{CAG\text{-}LSL\text{-}tdTomato/+}$ (*MyoD-tdTomato*) primed populations were initially labeled in myotome (M; Red). NT, neural tube; DRG, dorsal root ganglia; N, notochord; DML, dorsomedial lip; VLL, ventrolateral lip.

(B) Mouse *Pax3*, *MyoD*, and *Pax3+MyoD* infected mouse embryonic fibroblasts (MEFs) derived from *Pax3-GFP;MyoD-tdTomato* embryos after 7 days. Scale bars, 50 μm.

(C) FACS analyses with MEFs following the time course after *MyoD* infection, from day 3 to day 14.

Mesodermal Differentiation with *PAX3*$^{GFP/+}$ Human Induced PSCs

To identify PAX3-positive human MuSCs, we generated *Pax3*$^{GFP/+}$ knockin human induced PSCs (hiPSCs) using a BAC construct with a GFP insertion in the ATG start codon of the human *PAX3* gene (Figure S4A). Intrinsic GFP expression in cells derived from these hiPSCs could be detected in teratomas or neural precursors after SFEBq induction (GFP expressions in Figure 3A; Watanabe et al., 2005). After cell sorting with induced cells, GFP-positive cells were successfully immunostained with an anti-PAX3 antibody, while GFP-negative cells did not show the transcription of *PAX3* (Figure 3B). This hiPSC *PAX3*$^{GFP/+}$ knockin cell line was further genetically modified by the introduction of a DOX-inducible MYOD cassette using the piggyBac transposon system (Tanaka et al., 2013). To induce differentiated myogenic cells derived from these hiPSCs, we administered DOX continuously for at least a week (Figure S4B). This resulted in myogenic cell differentiation but GFP-positive cells were only rarely detected, in keeping with the result seen with mouse fibroblasts (Figure 1C and +3F++DOX in Figure 2C). However, transiently DOX-treated hiPSCs gave rise to significantly more GFP-positive cells than continuous or no DOX administration (DOX for 72 h in Figure 3C). To investigate gene expression in these GFP-positive cells we analyzed myogenic, neural, and neural crest markers, since *Pax3* is expressed in the corresponding cell types of developing embryos. GFP-positive cells

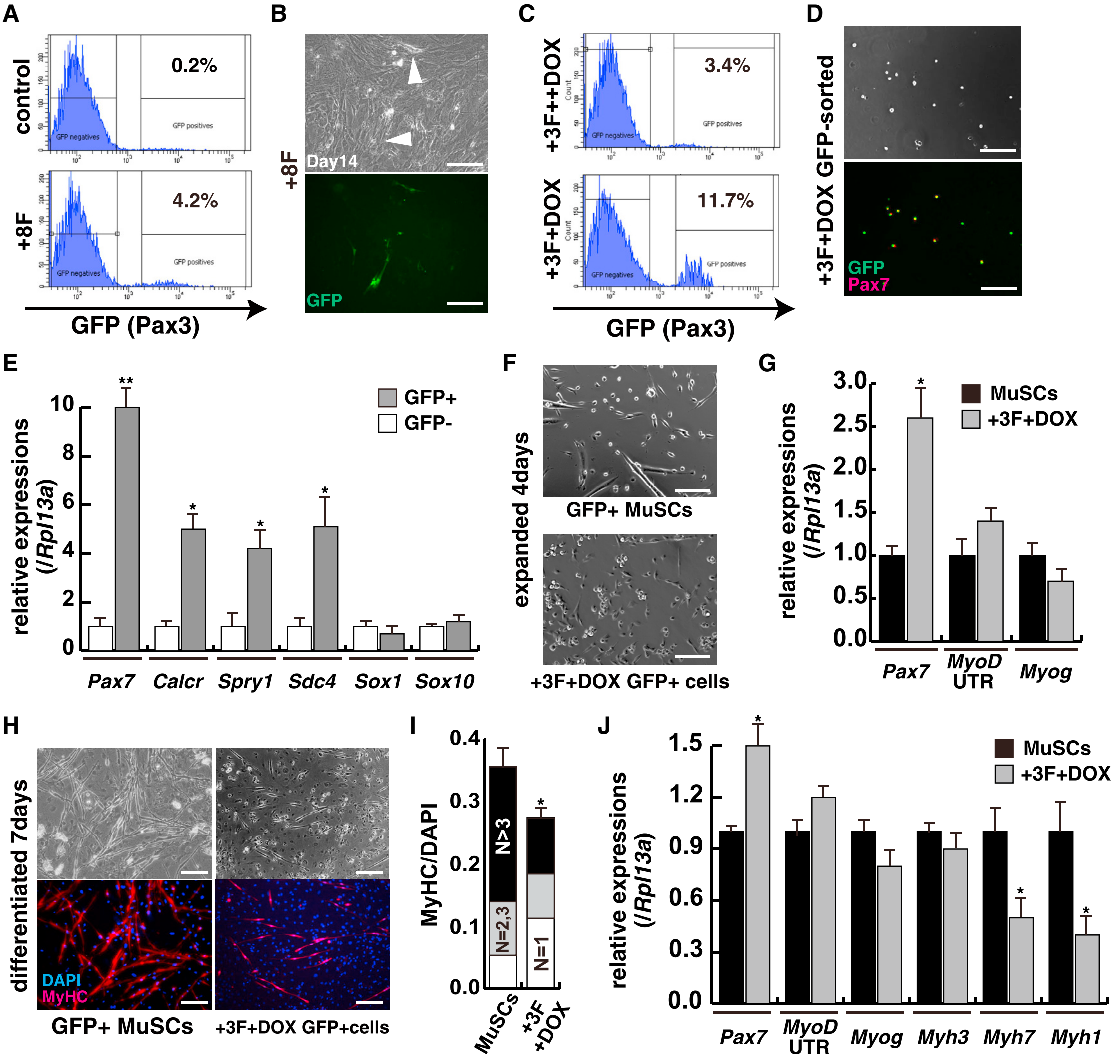

Figure 2. The Combination of Four Transcription Factors Induces PAX3-Expressing Myogenic Cells from Mouse Fibroblasts

(A and B) Detectable GFP expression in MEFs (arrowheads) infected with eight transcription factors (+8F) after 14 days.

(C) FACS analyses of GFP-expressing cells with mouse *Pax3*, *HeyL*, and *Klf4* (+3F) and persistent MYOD (++DOX) or transient MYOD for 72 h (+DOX).

(D) Immunofluorescence for GFP (labeled with Alexa 488, green) and Pax7 (labeled with Alexa 647, red) with GFP-positive cells induced by 3F and transient MYOD acceleration (+3F+DOX).

(E) Expression levels of *Pax7*, *Calcr*, *Spry1*, *Sdc4*, *Sox1*, and *Sox10* transcripts levels in induced Pax3-GFP-positive (gray) or -negative (white) cells with 3F+DOX. n = 3 independent replicates; p values are determined by t test from a two-tailed distribution. $^{**}p < 0.01$, $^{*}p < 0.05$.

(F) Morphological features of cultured GFP-positive satellite cells (GFP^{+} MuSCs; upper panel) and three transcription factors with transient DOX treatment (+3F+DOX GFP^{+} cells; lower panel) for 4 days.

(G) Myogenic transcripts of *Pax7*, endogenous *MyoD* (*MyoD UTR*), and *Myog* relative to Rpl13a transcripts in GFP^{+} cells cultured for 4 days. n = 3 independent replicates; p values are determined by t test from a two-tailed distribution. $^{*}p < 0.05$.

(H) Differentiated myogenic cells (DAPI, blue; MyHC, Alexa 647, red) from induced GFP-positive cells with 3F+DOX (right panels) compared with mouse Pax3-GFP satellite cells (GFP^{+} MuSCs; left panels) on 2% horse serum for 7 days.

(I) Quantification of the ratio of DAPI-positive mono (N = 1) or multiple nuclei (N = 2, 3 or N > 3) present in single MyHC-positive myofibers of (H). n = 3 independent replicates; p values are determined by t test from a two-tailed distribution. $^{*}p < 0.05$.

(J) Transcriptional levels of myogenic markers *Pax7*, *MyoD*, *Myog*, *Myh3*, *Myh7*, and *Myh1*, differentiated for 7 days from GFP^{+} cells. n = 3 independent replicates; p values are determined by t test from a two-tailed distribution. $^{*}p < 0.05$.

Error bars indicate ±SEM. Scale bars, 50 μm.

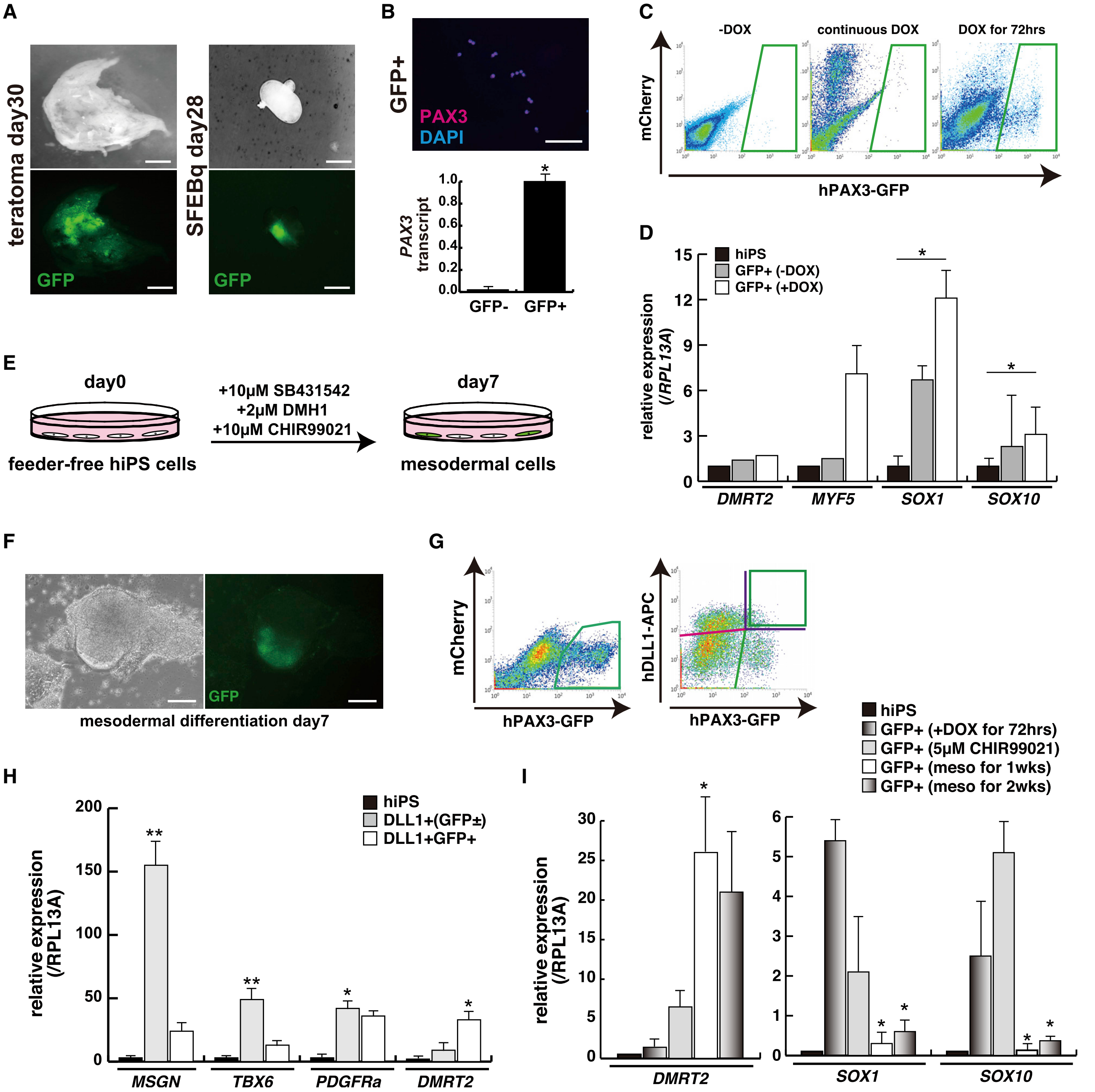

Figure 3. Mesodermal Differentiation from *PAX3*$^{GFP/+}$-Derived hiPSCs

(A) GFP expression in induced teratoma (left panels) and neural precursors by SFEBq induction (right panels) derived from *PAX3*$^{GFP/+}$ hiPSCs. Scale bars, 200 μm.

(B) Immunostaining with Pax3 antibody in GFP-sorted cells (upper panel) and the ratio of *PAX3* transcript relative to *RPL13*A in GFP-sorted cells (lower panel). Red, PAX3; blue, DAPI. Scale bar, 100 μm.

(C) FACS profiles of *Pax3*$^{GFP/+}$; *Tet-On MYOD* hiPSCs with or without continuous doxycycline (DOX) treatment (left panel, no DOX; middle panel, continuous DOX) and transient treatment for 72 h and cultured for a total of 7 days (right panel, DOX for 72 h).

(D) Relative transcription levels of dermomyotome (*DMRT2*), myogenic (*MYF5*), and neural (*SOX1*, *SOX10*) progenitors in induced GFP-positive cells treated with doxycycline for 72 h and cultured for a total of 7 days. n = 3 independent replicates; p values are determined by t test from a two-tailed distribution. *$p < 0.05$.

(E) Schematic representation of the induction to mesodermal cells from hiPSCs.

(F) Morphological features and GFP expression of mesodermal differentiated cells derived from *PAX3*$^{GFP/+}$;*Tet-On MYOD* hiPSCs.

(legend continued on next page)

derived from DOX-treated hiPSCs acquired myogenic potential, as shown by the presence of *MYF5* transcripts; however, neural transcripts of *SOX1* or *SOX10* were also detected (Figure 3D). This suggests that neural PAX3-positive cells may also arise even in the MuSC culture condition with transient MYOD expression (Figure S4C). We therefore attempted to exclude neural induction from hiPSCs by using modified cell-culture conditions to favor mesodermal, not ectodermal, lineages. Several groups have reported culture conditions that promote induction of somitic cells in the mesodermal lineage from human PSCs, and we adopted a method to induce paraxial mesodermal cells by manipulation of transforming growth factor β, bone morphogenetic protein, and Wnt signaling (Nakajima et al., 2018, Figure 3E). PAX3-GFP-expressing cells were obtained in induced aggregates of cells, reminiscent of mouse somites (Figure 3F), showing partial expression of DLL1, which marks the paraxial mesodermal lineage, during somite differentiation (green in Figure 3G). We evaluated induced $DLL1^+$ or $DLL1^+PAX3^+$ cells for expression of transcripts for *MESOGENIN* (*MSGN*), *TBX6*, and *PDGFRa*, which are presomitic mesodermal markers, or *DMRT2*, which is a dermomyotome marker of early somites (Sato et al., 2010). Successful mesodermal induction was demonstrated by high levels of *MSGN*, *TBX6*, or *PDGFRa* transcripts in total $DLL1^+$ cells, and high *DMRT2* expression in $DLL1^+GFP^+$ double-positive cells (Figure 3H). We next analyzed neural or non-neural transcripts in PAX3-GFP cells under different differentiation conditions. As shown in Figure 3I, on treatment with 5 μM CHIR99021, which promotes neural crest lineages derived from hiPSCs, including hiPSCs transiently treated with DOX for 72 h, the level of *SOX1* or *SOX10* transcripts was highly upregulated; however, GFP-positive cells treated with the mesodermal induction procedure for 1 week did not express neural crest markers but did express transcripts for *DMRT2*, also seen for a longer period of 2 weeks in culture (Figure 3I).

Additional Expression of Four Transcription Factors in Mesodermal Cells Induces PAX3-Positive Muscle Stem Cells

Using induced mesodermal cells labeled with DLL1, derived from hiPSCs, we attempted to determine which of the eight transcription factors was essential for the induction of PAX3-positive MuSCs (Figures 4A and S2). The combination of PAX3, HEYL, KLF4, and inducible transient MYOD (+3F+DOX) had the ability to induce PAX3-GFP (mCherry-positive/negative) cells effectively, in contrast to single transient or continuous MYOD (+DOX or +3F++DOX; Figures 4B and 4C). To evaluate whether these induced GFP cells have the characteristics of MuSCs, we checked cell-surface antigens, transcripts in these cells, and myogenic differentiation activity. Both CD56 and CD82, markers of myogenic stem cells and myocytes (Alexander et al., 2016), were co-expressed on 60% of 4F-induced Pax3-GFP cells (Figure 4D). These GFP-sorted cells showed mainly PAX7-positive staining; however, PAX7-negative cells were detected as MYOG-positive differentiated muscle cells (Figure 4E). When GFP-positive or -negative populations of CD56-positive myogenic cells were seeded under myogenic differentiation conditions with 2% horse serum, GFP-positive cells labeled the differentiation marker MYOG or MYHC, although only a few myofibers were observed (Figures 4F–4H) in comparison with GFP-negative cells of the $CD56^+$ population. This is consistent with downregulation of PAX3, and subsequently the GFP reporter on muscle differentiation and muscle fiber formation; the continuing presence of exogenous *PAX3* transcripts in this population of myogenic cells may explain why the number of myofibers is low (Figure 4I; Relaix et al., 2006). Comparison of transcript levels of *DMRT2*, *NFIX* that marks myogenic progenitors at fetal stages (Messina et al., 2010), or *NAP1L5* that marks adult MuSCs (Pallafacchina et al., 2010) shows that +3F+DOX leads to myogenic progenitor/stem cells of fetal and adult types (Figure 4J). Furthermore, the levels of *PAX7* or *CALCR* transcripts specifically expressed in adult quiescent MuSCs were high in induced GFP cells derived from mesodermal lineages compared with DOX-induced GFP cells (Figure 4K). In the absence of exogenous PAX3, the three factors HEYL, KLF4, and MYOD also induced PAX3-GFP cells from mesodermal cells; however *PAX7*, *CALCR*, or *NAP1L5* transcripts in GFP^+ cells were all decreased (Figure 5A), and the cells showed less myogenic differentiation (Figures 5B–5D). Comparison of the results obtained with induction by the four factors identified here with previously published protocols for inducing MuSCs, using growth factors (Chal et al., 2015) or with induced PAX7 (Darabi et al., 2012), showed that the protocol we describe leads to more mature MuSCs expressing adult satellite cell

(G) Cultured cell populations on mesodermal induction for 7 days (left panel) and subpopulation of ectodermal GFP (GFP^+DLL1^-) and mesodermal GFP (green box, GFP^+DLL^+) derived from presomitic mesoderm (GFP^-DLL1^+) labeled with the hDLL1 antibody (right panel).
(H) Transcription analyses of each type of sorted cells with presomitic mesodermal markers (*MSGN*, *TBX6*, *PCGFRa*) and the dermomyotome marker *DMRT2*. n = 3 independent replicates; p values are determined by Dunnett's multiple-comparisons test. $^*p < 0.05$, $^{**}p < 0.01$.
(I) Transcription analyses with PAX3-GFP-sorted cells on each differentiated condition. n = 3 independent replicates; p values are determined by Dunnett's multiple-comparisons test. $^*p < 0.05$, $^{**}p < 0.01$.
Error bars indicate ±SEM.

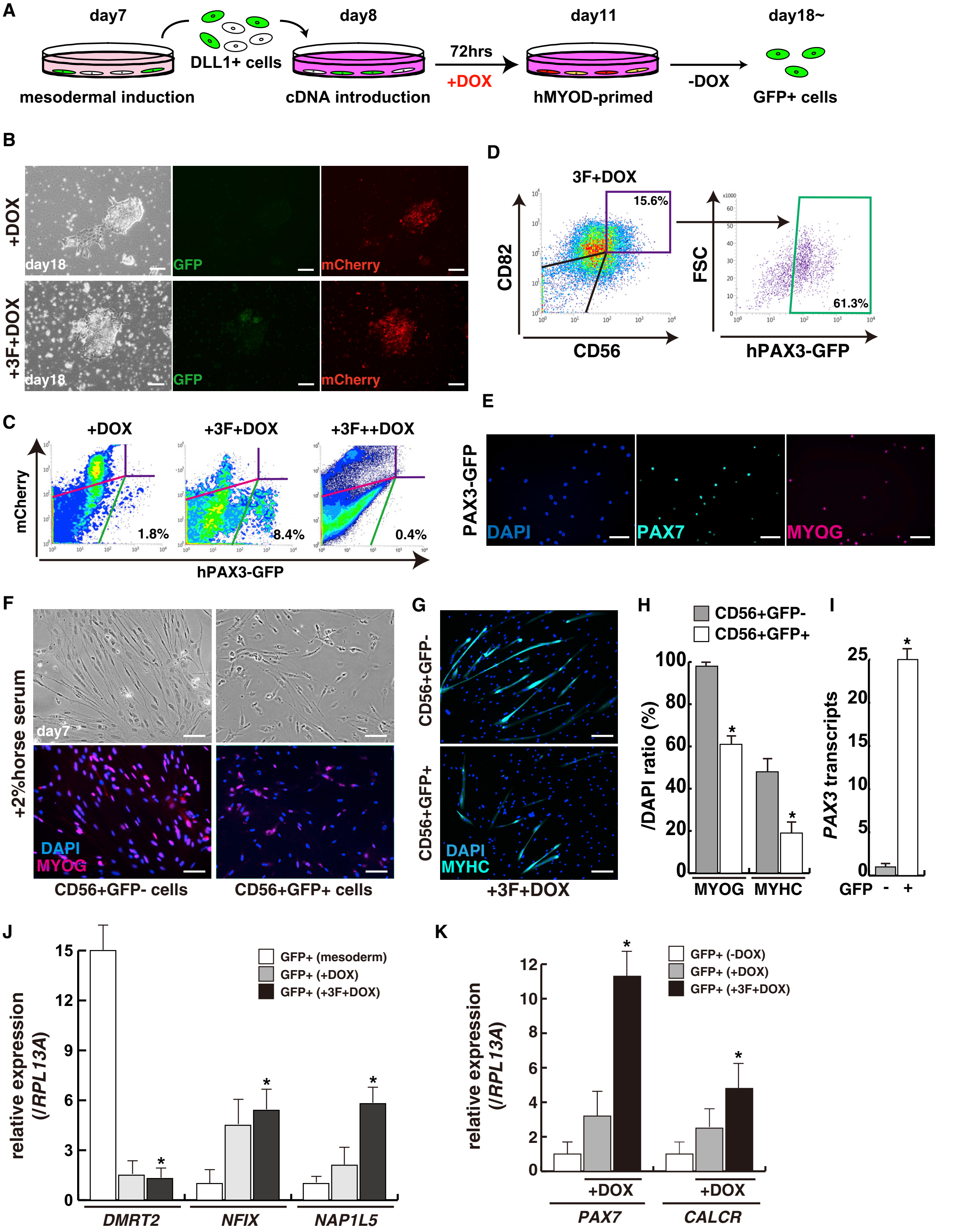

(legend on next page)

(legend on next page)

markers (Figure S5). We conclude that cells from mesodermal cells with the four transcription factors have the characteristics of skeletal MuSCs and show myogenic potential when cultured *in vitro*.

Transplanted PAX3-GFP Cells in the DMD Mouse Model Contribute to Skeletal Muscle Regeneration

Quiescent adult MuSCs are highly potent for skeletal muscle regeneration and form muscle fibers when they are transplanted into damaged muscle. We have previously reported that embryonic muscle precursors that are Pax3-GFP positive do not contribute effectively to regenerating muscle fibers in $Dmd^{-/y}$ mice (Sakai et al., 2013). We now tested the *in vivo* myogenic capacity of Pax3-GFP-positive cells obtained from hiPSCs by the three factors and transient MYOD procedure. $Dmd^{-/y}$;NSG (DMD) mice were engrafted with GFP^+ cells, which were directly collected after sorting to evaluate their MuSC regenerative capacity (Figure 6A). These GFP-positive cells effectively contributed to regenerated muscle fibers after their engraftment into DMD mice, as detected by human Dystrophin (DYS) expression in regenerating muscle fibers (mean = 282 DYS^+ fibers/section of the *tibialis anterior* (TA) muscle, 9.4% positives in total myofibers of the TA muscle, red in Figures 6B and 6C). When cultured for 4 days *ex vivo*, GFP-negative cells induced by the four factors (+cultured GFP^+ or +GFP^-, +3F+DOX), or GFP-positive cells induced by the four factors (GFP^-, +3F+DOX) or after DOX alone (+GFP^+, +DOX), these cells showed significantly reduced regenerative efficiency when the same number of cells was injected (0.43%, 0.60%, or 0.31%, respectively, Figure 6C). To investigate the capacity of PAX3-GFP-positive cells to renew the satellite cell compartment, we analyzed numbers of human-derived $PAX7^+$ cells underneath the basal lamina of mouse skeletal muscle fibers (arrowheads in Figure 6D). GFP-positive cells obtained after treatment with four factors occupied a satellite cell position on regenerated fibers, thus indicating renewal of the satellite cell compartment (mean = 27 $PAX7^+hLMNA^+$ cells/section, Figures 6D and 6E). In contrast, GFP-negative cells or other induced GFP cells, including MYOD-expressing cells induced by DOX alone, did not contribute to reconstitution of the satellite cell compartment (Figure 6E). Transplanted cells, which were induced by four factors, were detected in the position of muscle satellite cells and also in the interstitial area. Whereas cells in the satellite position did not show MYOD-positive signals (green arrowheads in left panel of Figure 6F), those in the interstitium were MYOD positive, consistent with their status as activated myogenic cells that are still contributing to regeneration (green arrowheads in right panel of Figure 6F). To understand the relationship between regeneration and cell expansion of transplanted cells, we found Ki67-positive cells in the transplanted area and non-proliferating transplanted cells on regenerated muscle fibers, which were DYS positive (red arrowheads in Figure 6G). Ki67-positive

Figure 4. Selected Four Transcription Factors Induce Human PAX3-Positive Muscle Stem Cells Derived from the Mesodermal Lineage

(A) Procedure for the induction of PAX3-GFP cells derived from mesodermal lineage.

(B) GFP or mCherry expression in transient MYOD-induced populations after expression of PAX3, HEYL, and KLF4 transcription factors (+3F), and doxycycline for 72 h (+DOX).

(C) The contrasting proportion of induced PAX3-GFP cells with transient single doxycycline treatment (+DOX) and three transcription factors and transient/continuous doxycycline (+3F+DOX/+3F++DOX).

(D) The FACS profile of CD56 and CD82 expression, human myogenic cell-surface markers, in induced cells. More than 60% of PAX3-GFP cells were merged with $CD56^+CD82^+$ as double positives.

(E) Immunostaining with anti-PAX7 (left panel, labeled with Alexa 488, light blue) and anti-MYOGENIN (MYOG; right panel, labeled with Alexa 647, purple) antibodies in $CD56^+CD82^+GFP^+$ sorted cells. GFP signals were not detected after immunostaining.

(F and G) *In vitro* myogenic differentiation assay with induced PAX3-GFP-positive ($CD56^+GFP^+$) or -negative ($CD56^+GFP^-$) cells of CD56-positive cells in 2% of horse serum medium for 7 days. MYOG, purple; MYHC, light blue (labeled with Alexa 647); DAPI, blue.

(H) Percentage of differentiated myogenic cells presented as the ratio of MYOG or MYHC/DAPI fluorescence. n = 3 independent replicates; p values are determined by t test from a two-tailed distribution. *$p < 0.01$.

(I) Ratio of transcripts of *PAX3* relative to *RPL13*A in myogenic differentiated cells from PAX3-GFP-positive (+) or -negative cells (−) of the $CD56^+$ population. n = 3 independent replicates; p values are determined by t test from a two-tailed distribution. *$p < 0.01$.

(J) Fetal (*NFIX*) and adult (*NAP1L5*) transcriptional analyses with PAX3-GFP-positive or -negative cells of CD56-positive cells induced by transient DOX treatment plus three transcription factors (+3F+DOX). Mesodermal GFP-expressing cells, marked by *DMRT2* (dermomyotome), were used as control (GFP^+ mesoderm). n = 3 independent replicates; p values are determined by Dunnett's multiple-comparisons test. *$p < 0.01$.

(K) Transcriptional analyses with GFP-positive cells induced by transient DOX treatment plus three transcription factors (+3F+DOX). GFP-expressing cells with or without DOX were used as control (GFP^+ −DOX/+DOX). n = 3 independent replicates; p values are determined by Dunnett's multiple-comparisons test. *$p < 0.01$.

Error bars indicate ±SEM. Scale bars, 50 μm.

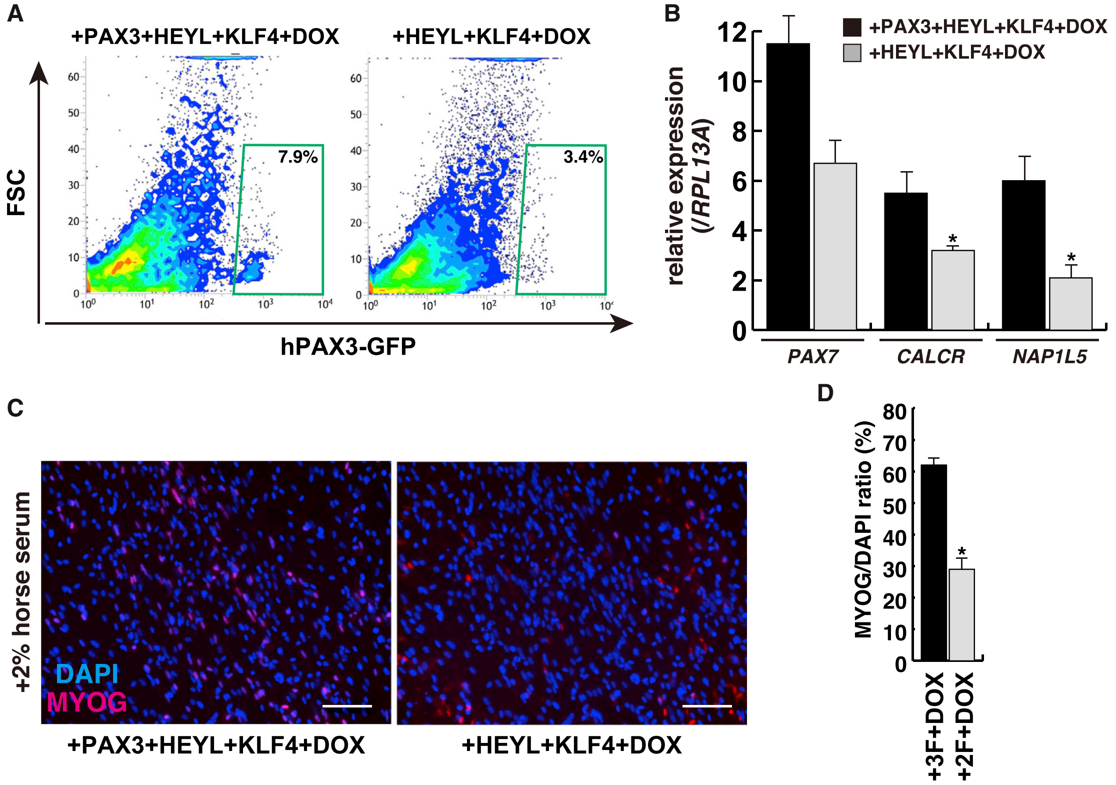

Figure 5. The Effect of Removing PAX3 from the Candidate Four Transcription Factors for Inducing PAX3-Expressing Muscle Stem Cells from hiPSCs

(A) FACS profile for detecting PAX3-GFP cells with or without PAX3 from mesodermal cells.

(B) Transcriptional analyses with GFP-positive cells induced by transient DOX treatment plus three transcription factors (+3F+DOX) or two transcription factors (no PAX3, +2F+DOX). n = 3 independent replicates; p values are determined by t test from a two-tailed distribution. $^*p < 0.01$.

(C) Myogenic differentiation with induced PAX3-GFP-positive cells in 2% of horse serum medium for 7 days. MYOG, purple (labeled with Alexa 647); DAPI, blue.

(D) Percentage of differentiated myogenic cells presented as MYOG/DAPI ratio. n = 3 independent replicates; p values are determined by t test from a two-tailed distribution. $^*p < 0.01$.

Error bars indicate ±SEM.

cells were detected in the interstitial area, not in the DYS-expressing area (white arrowhead in Figure 6G). We conclude that induced-PAX3 positive cells, grown under mesodermal culture conditions and expressing the four transcription factors, contribute to the formation of new skeletal muscle fibers, thus recapitulating aspects of normal regeneration after cell transplantation *in vivo*.

DISCUSSION

Here we define a combination of myogenic reprogramming factors that can induce MuSCs, capable of regenerating adult skeletal muscle. This is in contrast to MYOD, which converts non-muscle cells to the myogenic lineage, with the formation of differentiating myoblasts. However, these cells, which do not express PAX3 or PAX7, do not have the regenerative potential of adult MuSCs, as illustrated by our analysis of engraftment with mesodermal cells expressing MYOD alone or the effect of culturing MuSCs, which leads to the activation of MYOD expression with downregulation of PAX3 or PAX7 and subsequent loss of their capacity to repair injured muscle after engraftment (Montarras et al., 2005). Genetic tracing experiments have shown that most MuSCs are derived from cells that had expressed myogenic regulatory factors, MYOD, MYF5, or MRF4, and indeed still transcribe the *Myf5* gene; however the corresponding proteins are undetectable in adult MuSCs (Crist et al., 2012; Sato et al., 2014).

To test the effects of controlled MYOD activation on the induction of MuSCs, we used the Tet-On MYOD system. MYOD activation for over a week efficiently leads to

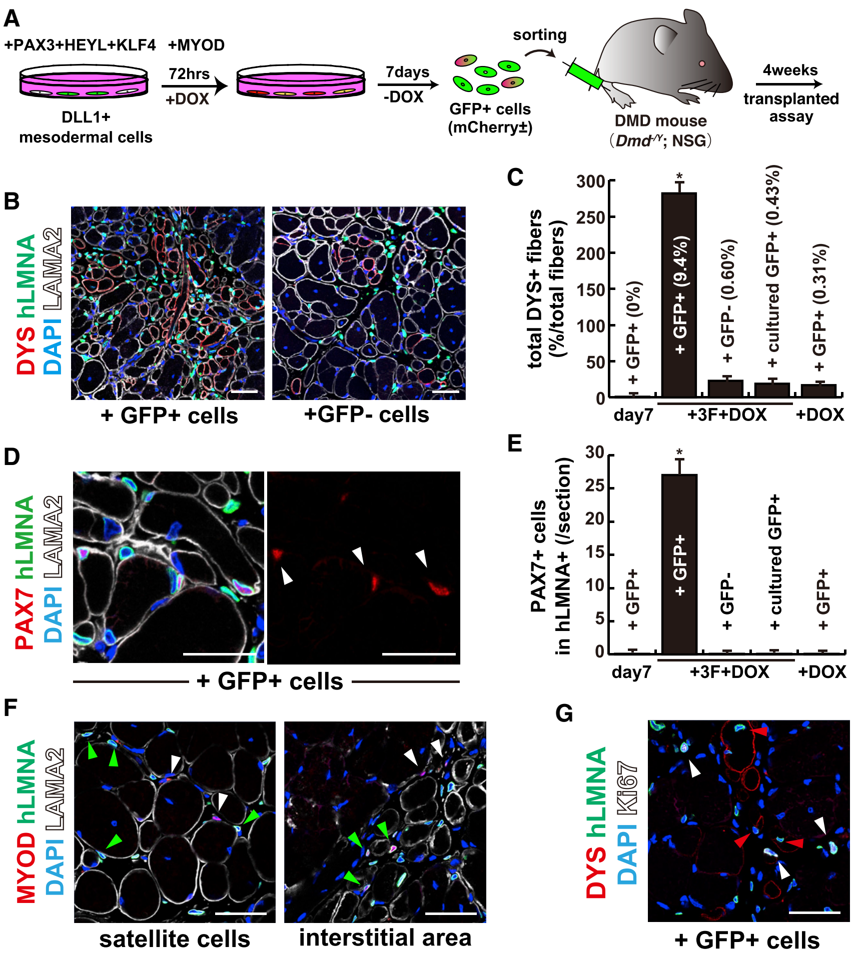

Figure 6. Induced Human PAX3-GFP Cells Have High Regenerative Capacity in Dystrophin-Deficient Mice

(A) Flowchart for the transplantation into $DMD^{-/y}$;NSG mice with PAX3-GFP-positive cells derived from human iPSCs.

(B) Comparison between the same number of GFP-positive and -negative cells (+GFP^{+} cells, +GFP^{-} cells) for transplantation. Immunostaining for dystrophin (DYS; labeled with Alexa 594, red), human nuclear lamin A/C (hLMNA; labeled with Alexa 488, green), laminin-a2 (LAMA2; labeled with Alexa 647, white), and DAPI (blue) on engrafted *tibialis anterior* (TA) muscle with 1.0 × 10^6 GFP-positive cells.

(C) Quantification of total DYSTROPHIN-positive (DYS+) regenerative myofibers on the section transplanted with equal numbers of GFP^{+} cells induced for 7 days as embryonic mesodermal cells (day 7), GFP-positive or -negative cells accelerated with four transcription factors (+3F+DOX cells), and culture for 4 days after sorting for GFP-positive cells induced by four transcription factors (cultured GFP^{+}) or by DOX treatment alone (+DOX). n = 3 independent replicates; p values are determined by Dunnett's multiple-comparisons test. *$p < 0.01$.

(D) Immunostaining for PAX7 (DYS; labeled with Alexa 594, red), human nuclear lamin A/C (hLMNA; labeled with Alexa 488, green), laminin-a2 (LAMA2; labeled with Alexa 647, white), and DAPI (blue) on a section of engrafted TA with +3F+DOX-induced PAX3-positive cells.

(E) The quantification of total PAX7;hLMNA double-positive cell numbers on a section of transplanted myofibers to contribute as MuSCs *in vivo* with cells as in (C). n = 3 independent replicates; p values are determined by Dunnett's multiple-comparisons test. *$p < 0.01$.

(F) Immunodetection with MYOD antibody (labeled with Alexa 594, red) on GFP-transplanted sections. MYOD-positive cells derived from mouse host (white arrowheads) and transplanted cells (green arrowheads) in the position of muscle satellite cells (left panel) and muscle interstitium (right panel).

(G) Ki67 staining on a section of engrafted TA with +3F+DOX-induced GFP cells. White arrowheads indicate Ki67-positive cells (labeled with Alexa 647). Red arrowheads have no Ki67 signals on regenerating myofibers labeled with DYSTROPHIN (DYS; labeled with Alexa 594, red) and human nuclear lamin A/C (hLMNA; labeled with Alexa 488, green).

Error bars indicate ±SEM. Scale bars, 50 μm.

terminally differentiated skeletal muscle cells derived from hiPSCs (Figure S4B); however, transient induction of MYOD expression led to the presence of *PAX3*-expressing cells and the absence of differentiated cells marked by skeletal muscle MyHC. In these cultured cells, we detected not only myogenic but also neural cells in the presence of persistent MYOD activation under conditions of high fetal bovine serum (FBS), suitable for *ex vivo* expansion of myogenic cells (Figure S4C). Transient MYOD activation and exogenous factors in the serum appear not to prevent some non-skeletal muscle derivatives from iPSCs, although MYOD might be essential for the induction of MuSCs from mouse fibroblasts as previously reported (Bar-Nur et al., 2018). Non-myogenic neural derivatives were excluded in these experiments when hiPSCs were cultured under medium conditions that favor mesoderm. In these conditions, early somite markers were observed. In the experiments described here, we employed the same hiPSC line to generate two subclones, which have different copy numbers of MYOD transgenes after treatment with the piggyBac transposon system, and these were used for the induction of PAX3-positive cells. Results with other hiPSC

lines, together with a different gene integration system to control MYOD levels, will be checked in further investigations.

In our investigation to identify regulatory factors common to MuSCs during development and in the adult, we identified four factors that promote the formation of this cell type from mouse and also human fibroblasts, and from hiPSCs. Moreover we show that this induction method leads to more mature MuSCs compared with previously reported methods to induce PAX3-positive myogenic precursor cells from hiPSCs with several growth and chemical factors (Chal et al., 2015) or induced PAX7 expression in human embryonic stem cells/hiPSCs after the formation of embryoid bodies (Darabi et al., 2012). This is illustrated by high levels of transcripts of *CALCR* or *NAP1L5*, which mark adult MuSCs under the conditions we describe with HEY, KLF4, PAX3, and transient MYOD and which we have not detected with the other reported induction methods.

MYOD, although unable to promote the formation of Pax3-GFP-positive MuSCs when overexpressed on its own, had a positive effect in combination with other factors. This is perhaps surprising but might reflect its role as a chromatin remodeler (de la Serna et al., 2005), facilitating the function of the other factors that more specifically render upstream myogenic factors accessible for transcription. *Pax3* and *Pax7* genes continue to be expressed in myoblasts and are only downregulated on muscle cell differentiation (Young and Wagers, 2010).

One of the four transcription factors, HEYL, is involved in Notch signaling. This signaling directly targets *Hes1*, *Hey1*, or *HeyL*, known to be highly expressed in quiescent MuSCs and essential for the generation of adult MuSCs, to suppress *MyoD* transcription and to regulate myogenic differentiation (Fukada et al., 2011). It has been reported that interstitial PAX7-positive myogenic stem cells have the ability to traverse the basal lamina and locate in the interstitial region, and that this process is dependent on Notch signaling (Bröhl et al., 2012). Therefore, transplanted cells affected by Notch signaling may be detected as activated myogenic cells in the interstitium, although the reason why and how $Pax7^{+}$ MuSCs come to be located outside the basal lamina are poorly understood. Another of the factors, KLF4, a member of the family of Kruppel-like factors, is known as a reprogramming factor that can induce PSCs (Takahashi and Yamanaka, 2006). This factor was chosen for the MuSC transcriptome screening because the KLF family is essential for the maintenance of MuSCs (Hayashi et al., 2016).

Exogenous PAX3 and/or PAX7 expression in PSCs has been proposed to enable efficient propagation of myogenic progenitors and has been used to produce myogenic stem cells for engraftment (Darabi et al., 2012; Kim et al., 2017). We have found that the three factors HEYL, KLF4, and MYOD, without exogenous PAX3 also induced PAX3-GFP cells from mesodermal cells; however, *PAX7*, *CALCR*, or *NAP1L5* transcripts in GFP^{+} cells without exogenous PAX3 were all decreased and showed less myogenic differentiation *in vitro*. Furthermore, additional expression of PAX3/PAX7 is probably important for the long-term maintenance of MuSCs (Buckingham and Relaix, 2015), and the activation of transient PAX3 promotes *ex vivo* expansion of mouse PAX7-positive MuSCs (Filareto et al., 2015). Manipulation of culture medium by itself, without the introduction of expression vectors, is being used to promote the formation of myogenic cells for cell therapy (Chal et al., 2015), although the addition of the four factors that we describe increased the production of MuSCs and also conferred more adult MuSC-like properties as discussed. However, in culture these PAX3-GFP cells did not all differentiate, probably because of high levels of PAX3 in some cells that prevent the normal downregulation of the protein required for the efficient onset of myogenic differentiation. Furthermore, the PAX3-GFP-expressing cells are not probably forming mature muscle on *in vitro* differentiation, as indicated by the MyHC isoforms expressed, some of which require innervation. *Pax3* expression marks all myogenic progenitors in the early embryo, and continues to be transcribed in a subset of adult MuSCs (Relaix et al., 2005, 2006) but not in the hind limbs, including the TA muscles. In our experiments, GFP signals were not detected in mouse TA muscles after transplantation with induced PAX3-expressing cells, suggesting that *Pax3* expression continues to be downregulated in mouse limbs (Boutet et al., 2012) and thus will not interfere with *in vivo* myogenic differentiation of transplanted cells.

Obtaining mature muscle fibers in culture presents another challenge. The important point for any therapeutic application to muscular disorders is that candidate muscle stem cells should be capable of behaving like intact adult MuSCs in an adult skeletal muscle environment, as we show using the induced cells derived from fibroblasts and hiPSCs described here. Further investigation would be required to understand the cellular and molecular mechanisms of these transcription factors to induce MuSCs, and in order for them to be used as valuable sources for stem cell therapies.

EXPERIMENTAL PROCEDURES

Mice

All animal experiments were approved by the Ethics Committee of Animal Experimentation of Kyoto Prefectural University of Medicine (permission number M26-237). All injections with needles were performed under anesthesia, and all efforts were made to minimize suffering. $Pax3^{GFP/+}$, *MyoD-CreIZ*, and

Rosa26$^{CAG\text{-}LSL\text{-}tdTomato/+}$ mice were used for obtaining skeletal muscle cells, and female *Dmd* $^{-/y}$ crossed with male NSG mice (Sato et al., 2014, Charles River) were used for the transplanted donors. Male *Dmd* $^{-/y}$;NSG mice were used for all experiments at the indicated ages.

Eight-week-old *Dmd* $^{-/y}$;NSG host male mice were used for engraftment of freshly isolated or cultured cells derived from hiPSCs (5.0×10^5 cells per 20 μL of PBS with 2% horse serum [Sigma]), and 10 μM Y-27632 (Nacalai Tesque) into TA muscle fibers. Mice were anesthetized with diethyl ether prior to engraftment. TA muscle was removed 4 weeks after transplantation, fixed, and stained as described below. Three independent mice for the transplantation and ten sections of each transplanted mouse were analyzed.

cDNA Constructs

For overexpression in mouse fibroblasts, pMXs-Gateway vector (Addgene #18656) was used. All coding sequences of selected 38 mouse genes were amplified by RT-PCR with KOD FX *Neo* DNA polymerase (Toyobo) and single strand cDNA synthesized by SuperScript 3 Reverse Transcriptase (Invitrogen) from mouse Pax3-GFP adult MuSCs. These cDNAs were subsequently subcloned into the pENTR1 and pMXs vectors. For human iPSCs, piggyBac-Gateway vector was used (Addgene #20960). All human cDNA constructs involving coding sequences were obtained from the human proteome expression resource HuPEX library (Goshima et al., 2008). Human cDNAs were transferred into the pLVX vector (Clontech) to overexpress in human fibroblasts and the pPiggyBac-Gateway vector with the LR clonase (Invitrogen) to overexpress in human iPSCs.

Cell Sorting

For live cell sorting and culture, skeletal muscle of limbs and diaphragms of embryonic-day-16.5 embryos, diaphragms of 1-week-old mice at a juvenile stage, and 12-week-old mice at an adult stage were dissected into small pieces and treated with 0.2% of collagenase (Worthington) in DMEM (Nacalai Tesque) at 37°C for 30 min to a few hours until the tissues disappeared. Dissociated cell suspensions were centrifuged and resuspended with 1% BSA (Sigma) in PBS stained with SYTOX Green or Red Dead Cell Stain (Molecular Probes) to exclude dead cells. For mesodermal cell sorting derived from human iPSCs, APC-conjugated anti-DLL1 antibody was used. Cell sorting and analyses were performed with FACSJazz and FACSAria3 (Becton Dickinson).

Cell Culture

Isolated embryonic fibroblasts of *Pax3-GFP*;*MyoD-tdTomato* mouse, or human primary dermal fibroblasts (Lonza) were expanded onto coated dishes with 0.1% gelatin, and 10% FBS (Gibco) in DMEM. Two independent human iPSC lines (#11-8 and #11-19 *PAX3*$^{GFP/+}$;Tet-*MYOD*) were cultured on iMatrix (Nippi)-coated dishes in StemFit AK02 medium (Ajinomoto). Cells were passaged as single cells. To induce PAX3-positive cells, we carried out the initial procedure on single cells plated on Geltrex-coated (Gibco) 6-well plates (5×10^4 cells/well) in mTeSR medium (STEMCELL Technologies) supplemented with 10 μM Y-27632 for 3 days. At day 3, culture medium was changed to mesodermal differentiation medium (50% Iscove's modified Dulbecco's medium and 50% F12 supplemented with 1% BSA, 1% CD lipid concentrate [Gibco], 1% insulin-transferrin-selenium [GIBCO], 20 μM 1-thioglycerol, 10 μM SB431542 [StemGent], 10 μM CHIR99021 [StemGent], 2 μM DMH1 (Tocris), and 20 ng/mL basic fibroblast growth factor [FGF; Wako]) for 4 days. At day 7, cells were dissociated with TrypLE select (Gibco) and mesodermal PAX3-GFP positives were selected by the cell sorter as DLL1-positive cells, electroporated by NEPA21 (Nepagene), mixed with 1.0×10^6 cells and 5 μg of plasmid DNAs, as described in Tanaka et al. (2013), and seeded onto Geltrex-coated dishes in DMEM/F12 medium supplemented with 20% FBS and 10 ng/mL basic FGF (MuSC medium) for 24 h. At day 8, culture medium was changed to MuSC medium supplemented with 1 ng/mL doxycycline (DOX; Tocris) for 3 days. After DOX treatment, culture medium was changed to MuSC medium for 2 weeks. The observation of cultured cells was performed by BZ-X710 microscopy (Keyence).

Microarray Analyses

The datasets for gene expression in mouse Pax3-GFP-expressing cells (embryonic day 16.5, 1 week old, 12 weeks old) and other stem cells were obtained from the NCBI GEO database (GEO: GSE11274 and GSE15155 [Ko et al., 2009; Pallafacchina et al., 2010]). All of the obtained Affymetrix CEL files were imported into GeneSpring GX (Agilent Technologies) for the calculation of expression levels and principal component analysis. Expression values were calculated with the GC-RMA summarization algorithm as implemented in GeneSpring GX software.

qRT-PCR Analyses

Total RNAs from sorted or cultured cells were extracted using an RNeasy Micro Kit (Qiagen). For qPCR analyses, synthesized cDNAs were prepared using SuperScript VILO MasterMix (Invitrogen). All qRT-PCR reactions were carried out in triplicate using Thunder Bird SYBR qPCR Mix (Toyobo) and Thermal Cycler Dice Realtime System (Takara), and normalized to mRNA expression level of mouse or human ribosomal protein L13A as a control. Primer sequences (5′ to 3′) are listed in Table S3.

Immunofluorescence

Cells and skeletal muscle tissues were fixed with 4% paraformaldehyde in PBS for 15 min at 4°C, prior to embedding in Frozen Section Compound (Leica Microsystems) for cryosections. Fixed samples were incubated with 0.1%Triton X-100 in PBS for 5 min, BlockingOne (Nakacai Tesque) for 30 min, and anti-GFP (Molecular Probes A6455; diluted 1:500), anti-PAX3 (DSHB AB528426; diluted 1:100), anti-PAX7 (DSHB AB528428; diluted 1:100), anti-myogenin (DAKO M3559; diluted 1:100), anti-MyoD (Abcam ab64159; diluted 1:500), anti-laminin (Enzo Life Sciences ALX-804-190; diluted 1:500), anti-dystrophin (Abcam ab15277; diluted 1:200), anti-MyHC (R&D MAB4470; MF20, diluted 1:200), anti-FSP1 (Abcam ab124805; diluted 1:200), anti-lamin A/C (Abcam ab8984; diluted 1:200), and anti-Ki67 (Abcam ab15580; diluted 1:500) antibodies in 5% BlockingOne overnight at 4°C. After three washes with 0.1% Tween 20 in PBS, cells were incubated with Alexa-conjugated anti-mouse immunoglobulin G1 (IgG1), mouse IgG2b, rabbit IgG, or rat IgG antibodies (Molecular Probes; diluted

1:500). Cells were washed and mounted in ProLong Diamond antifade reagent with DAPI (Molecular Probes). Images were collected and processed by BZ-X710 microscopy.

Statistics

We report the statistical data including results of at least three biological replicates. Statistical analyses were performed with StatPlus software (AnalystSoft) to determine significant differences from a two-tailed distribution using a paired or unpaired Student's t test. The statistical significance for the comparison of multiple sample sets was determined with Dunnett's multiple comparisons following ANOVA. p values are indicated on each figure as <0.05 (*) and <0.01 (**). All error bars represent mean ± SD.

SUPPLEMENTAL INFORMATION

Supplemental Information can be found online at https://doi.org/10.1016/j.stemcr.2019.06.006.

AUTHOR CONTRIBUTIONS

T.S. conceived and designed the experiments. T.S., K.H., H.S., T.Y., and N.G. performed the experiments and analyzed data. T.S., H.S., M.U., and C.S. contributed reagents, material, and analysis tools. T.S. and H.S. wrote the paper. T.S. edited the manuscript.

ACKNOWLEDGMENTS

We thank Dr. Nicholas Boyd-Gibbins for critical reading of the manuscript. This work was supported by grants-in-aid from the Ministry of Education, Culture, Sports, Science and Technology (MEXT, KAKEN#26870285, T.S.), Japan Agency for Medical Research and Development (AMED, H.S. and T.S.), Projects for Technology Development, Development of Cell Transplantation Methods for Refractory Muscle Diseases, and AMED-CREST (AMED, T.S.), and a grant from Nakatomi Memorial Foundation (T.S.).

Received: October 27, 2018
Revised: June 14, 2019
Accepted: June 25, 2019
Published: July 25, 2019

REFERENCES

Alexander, M.S., Rozkalne, A., Colletta, A., Spinazzola, J.M., Johnson, S., Rahimov, F., Meng, H., Lawlor, M.W., Estrella, E., Kunkel, L.M., et al. (2016). CD82 is a marker for prospective isolation of human muscle satellite cells and is linked to muscular dystrophies. Cell Stem Cell *19*, 800–807.

Bar-Nur, O., Gerli, M.F.M., Di Stefano, B., Almada, A.E., Galvin, A., Coffey, A., Huebner, A.J., Feige, P., Verheul, C., Cheung, P., et al. (2018). Direct reprogramming of mouse fibroblasts into functional skeletal muscle progenitors. Stem Cell Reports *10*, 1505–1521.

Boutet, S.C., Cheung, T.H., Quach, N.L., Liu, L., Prescott, S.L., Edalati, A., Iori, K., and Rando, T.A. (2012). Alternative polyadenylation mediates microRNA regulation of muscle stem cell function. Cell Stem Cell *10*, 327–336.

Bröhl, D., Vasyutina, E., Czajkowski, M.T., Griger, J., Rassek, C., Rahn, H.P., Purfürst, B., Wende, H., and Birchmeier, C. (2012). Colonization of the satellite cell niche by skeletal muscle progenitor cells depends on Notch signals. Dev. Cell *23*, 469–481.

Buckingham, M., and Relaix, F. (2015). PAX3 and PAX7 as upstream regulators of myogenesis. Semin. Cell Dev. Biol. *44*, 115–125.

Cerletti, M., Jurga, S., Witczak, C.A., Hirshman, M.F., Shadrach, J.L., Goodyear, L.J., and Wagers, A.J. (2008). Highly efficient, functional engraftment of skeletal muscle stem cells in dystrophic muscles. Cell *134*, 37–47.

Chal, J., Oginuma, M., Al Tanoury, Z., Gobert, B., Sumara, O., Hick, A., Bousson, F., Zidouni, Y., Mursch, C., Moncuquet, P., et al. (2015). Differentiation of pluripotent stem cells to muscle fiber to model Duchenne muscular dystrophy. Nat. Biotechnol. *33*, 962–969.

Collins, C.A., Olsen, I., Zammit, P.S., Heslop, L., Petrie, A., Partridge, T.A., and Morgan, J.E. (2005). Stem cell function, self-renewal, and behavioral heterogeneity of cells from the adult muscle satellite cell niche. Cell *122*, 289–301.

Crist, C.G., Montarras, D., and Buckingham, M. (2012). Muscle satellite cells are primed for myogenesis but maintain quiescence with sequestration of Myf5 mRNA targeted by microRNA-31 in mRNP granules. Cell Stem Cell *11*, 118–126.

Darabi, R., Arpke, R.W., Irion, S., Dimos, J.T., Grskovic, M., Kyba, M., and Perlingeiro, R.C. (2012). Human ES- and iPS-derived myogenic progenitors restore DYSTROPHIN and improve contractility upon transplantation in dystrophic mice. Cell Stem Cell *10*, 610–619.

de la Serna, I.L., Ohkawa, Y., Berkes, C.A., Bergstrom, D.A., Dacwag, C.S., Tapscott, S.J., and Imbalzano, A.N. (2005). MyoD targets chromatin remodeling complexes to the myogenin locus prior to forming a stable DNA-bound complex. Mol. Cell. Biol. *25*, 3997–4009.

Filareto, A., Rinaldi, F., Arpke, R.W., Darabi, R., Belanto, J.J., Toso, E.A., Miller, A.Z., Ervasti, J.M., McIvor, R.S., Kyba, M., et al. (2015). Pax3-induced expansion enables the genetic correction of dystrophic satellite cells. Skelet. Muscle *5*, 36.

Fukada, S., Uezumi, A., Ikemoto, M., Masuda, S., Segawa, M., Tanimura, N., Yamamoto, H., Miyagoe-Suzuki, Y., and Takeda, S. (2007). Molecular signature of quiescent satellite cells in adult skeletal muscle. Stem Cells *25*, 2448–2459.

Fukada, S., Yamaguchi, M., Kokubo, H., Ogawa, R., Uezumi, A., Yoneda, T., Matev, M.M., Motohashi, N., Ito, T., Zolkiewska, A., et al. (2011). Hesr1 and Hesr3 are essential to generate undifferentiated quiescent satellite cells and to maintain satellite cell numbers. Development *138*, 4609–4619.

Goshima, N., Kawamura, Y., Fukumoto, A., Miura, A., Honma, R., Satoh, R., Wakamatsu, A., Yamamoto, J., Kimura, K., Nishikawa, T., et al. (2008). Human protein factory for converting the transcriptome into an in vitro-expressed proteome. Nat. Methods *5*, 1011–1017.

Gros, J., Manceau, M., Thomé, V., and Marcelle, C. (2005). A common somitic origin for embryonic muscle progenitors and satellite cells. Nature *435*, 954–958.

Guiraud, S., Aartsma-Rus, A., Vieira, N.M., Davies, K.E., van Ommen, G.J., and Kunkel, L.M. (2015). The pathogenesis and therapy of muscular dystrophies. Annu. Rev. Genomics Hum. Genet. *16*, 281–308.

Hayashi, S., Manabe, I., Suzuki, Y., Relaix, F., and Oishi, Y. (2016). Klf5 regulates muscle differentiation by directly targeting muscle-specific genes in cooperation with MyoD in mice. Elife *5*, e17462.

Kim, J., Magli, A., Chan, S.S.K., Oliveira, V.K.P., Wu, J., Darabi, R., Kyba, M., and Perlingeiro, R.C.R. (2017). Expansion and purification are critical for the therapeutic application of pluripotent stem cell-derived myogenic progenitors. Stem Cell Reports *9*, 12–22.

Ko, K., Tapia, N., Wu, G., Kim, J.B., Bravo, M.J., Sasse, P., Glaser, T., Ruau, D., Han, D.W., Greber, B., et al. (2009). Induction of pluripotency in adult unipotent germline stem cells. Cell Stem Cell *5*, 87–96.

Messina, G., Biressi, S., Monteverde, S., Magli, A., Cassano, M., Perani, L., Roncaglia, E., Tagliafico, E., Starnes, L., Campbell, C.E., et al. (2010). Nfix regulates fetal-specific transcription in developing skeletal muscle. Cell *140*, 554–566.

Montarras, D., Morgan, J., Collins, C., Relaix, F., Zaffran, S., Cumano, A., Partridge, T., and Buckingham, M. (2005). Direct isolation of satellite cells for skeletal muscle regeneration. Science *309*, 2064–2067.

Morris, S.A. (2016). Direct lineage reprogramming via pioneer factors; a detour through developmental gene regulatory networks. Development *143*, 2696–2705.

Nakajima, T., Shibata, M., Nishio, M., Nagata, S., Alev, C., Sakurai, H., Toguchida, J., and Ikeya, M. (2018). Modeling human somite development and fibrodysplasia ossificans progressiva with induced pluripotent stem cells. Development *145*, dev165431.

Negroni, E., Bigot, A., Butler-Browne, G.S., Trollet, C., and Mouly, V. (2016). Cellular therapies for muscular dystrophies: frustrations and clinical successes. Hum. Gene Ther. *27*, 117–126.

Pallafacchina, G., François, S., Regnault, B., Czarny, B., Dive, V., Cumano, A., Montarras, D., and Buckingham, M. (2010). An adult tissue-specific stem cell in its niche: a gene profiling analysis of in vivo quiescent and activated muscle satellite cells. Stem Cell Res. *4*, 77–91.

Relaix, F., Rocancourt, D., Mansouri, A., and Buckingham, M. (2005). A Pax3/Pax7-dependent population of skeletal muscle progenitor cells. Nature *435*, 948–953.

Relaix, F., Montarras, D., Zaffran, S., Gayraud-Morel, B., Rocancourt, D., Tajbakhsh, S., Mansouri, A., Cumano, A., and Buckingham, M. (2006). Pax3 and Pax7 have distinct and overlapping functions in adult muscle progenitor cells. J. Cell Biol. *172*, 91–102.

Sakai, H., Sato, T., Sakurai, H., Yamamoto, T., Hanaoka, K., Montarras, D., and Sehara-Fujisawa, A. (2013). Fetal skeletal muscle progenitors have regenerative capacity after intramuscular engraftment in dystrophin deficient mice. PLoS One *8*, e63016.

Sato, T., Rocancourt, D., Marques, L., Thorsteinsdóttir, S., and Buckingham, M. (2010). A Pax3/Dmrt2/Myf5 regulatory cascade functions at the onset of myogenesis. PLoS Genet. *6*, e1000897.

Sato, T., Yamamoto, T., and Sehara-Fujisawa, A. (2014). miR-195/497 induce postnatal quiescence of skeletal muscle stem cells. Nat. Commun. *5*, 4597.

Shea, K.L., Xiang, W., LaPorta, V.S., Licht, J.D., Keller, C., Basson, M.A., and Brack, A.S. (2010). Sprouty1 regulates reversible quiescence of a self-renewing adult muscle stem cell pool during regeneration. Cell Stem Cell *6*, 117–129.

Takahashi, K., and Yamanaka, S. (2006). Induction of pluripotent stem cells from mouse embryonic and adult fibroblast cultures by defined factors. Cell *126*, 663–676.

Tanaka, A., Woltjen, K., Miyake, K., Hotta, A., Ikeya, M., Yamamoto, T., Nishino, T., Shoji, E., Sehara-Fujisawa, A., Manabe, Y., et al. (2013). Efficient and reproducible myogenic differentiation from human iPS cells: prospects for modeling Miyoshi myopathy in vitro. PLoS One *8*, e61540.

Watanabe, K., Kamiya, D., Nishiyama, A., Katayama, T., Nozaki, S., Kawasaki, H., Watanabe, Y., Mizuseki, K., and Sasai, Y. (2005). Directed differentiation of telencephalic precursors from embryonic stem cells. Nat. Neurosci. *8*, 288–296.

Weintraub, H., Tapscott, S.J., Davis, R.L., Thayer, M.J., Adam, M.A., Lassar, A.B., and Miller, A.D. (1989). Activation of muscle-specific genes in pigment, nerve, fat, liver, and fibroblast cell lines by forced expression of MyoD. Proc. Natl. Acad. Sci. U S A *86*, 5434–5438.

Young, A.P., and Wagers, A.J. (2010). Pax3 induces differentiation of juvenile skeletal muscle stem cells without transcriptional upregulation of canonical myogenic regulatory factors. J. Cell Sci. *123*, 2632–2639.

Stem Cell Reports, Volume *13*

Supplemental Information

Core Transcription Factors Promote Induction of PAX3-Positive Skeletal Muscle Stem Cells

Takahiko Sato, Koki Higashioka, Hidetoshi Sakurai, Takuya Yamamoto, Naoki Goshima, Morio Ueno, and Chie Sotozono

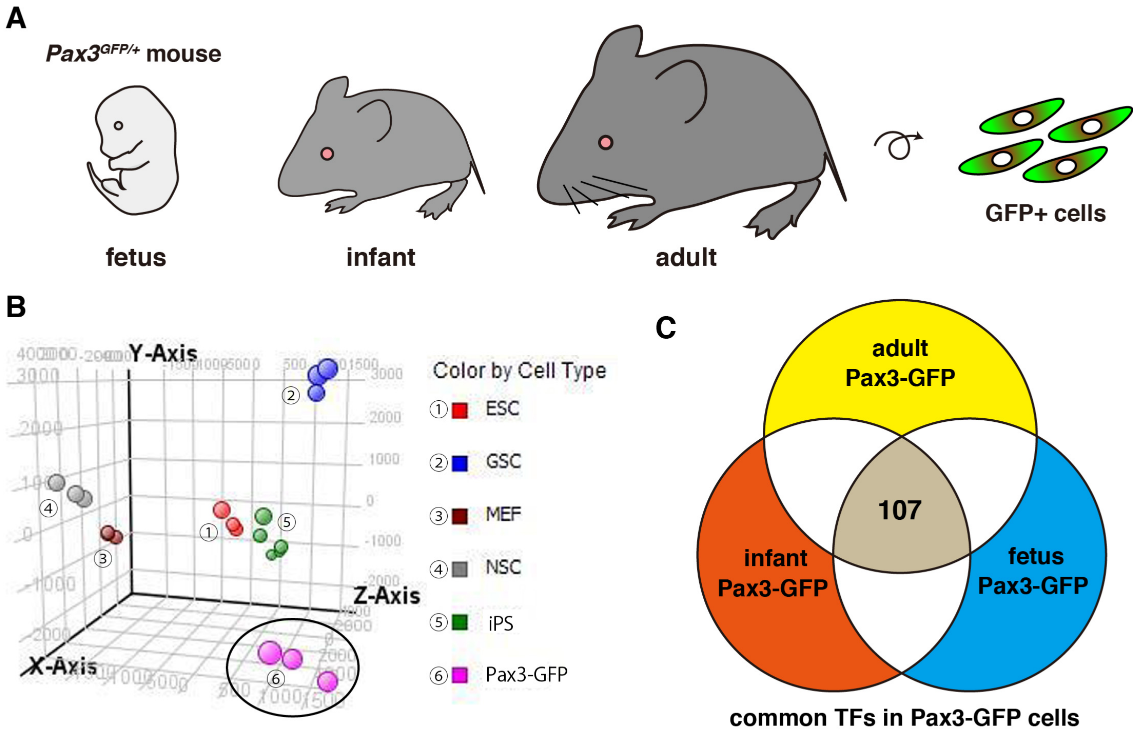

Figure S1. Gene profile from myogenic precursors and adult muscle stem cells of Pax3$^{GFP/+}$ mice.

(A) 3 different developmental stages (fetus, infant, and adult mouse) were used for isolating Pax3-GFP expressing cells of skeletal muscle tissue.

(B) Principal component analysis (PCA) with Pax3-GFP cells and other types of cells including ESC (mouse embryonic stem cells), GSC (mouse germline stem cells), MEF (mouse embryonic fibroblasts), NSC (mouse neural stem cells), and iPS (mouse induced pluripotent stem cells).

(C) 107 selected genes, which are highly expressed in Pax3-expressing cells derived from 3 different stages of *Pax3*$^{GFP/+}$ mice, relative to other cells (see Table S1).

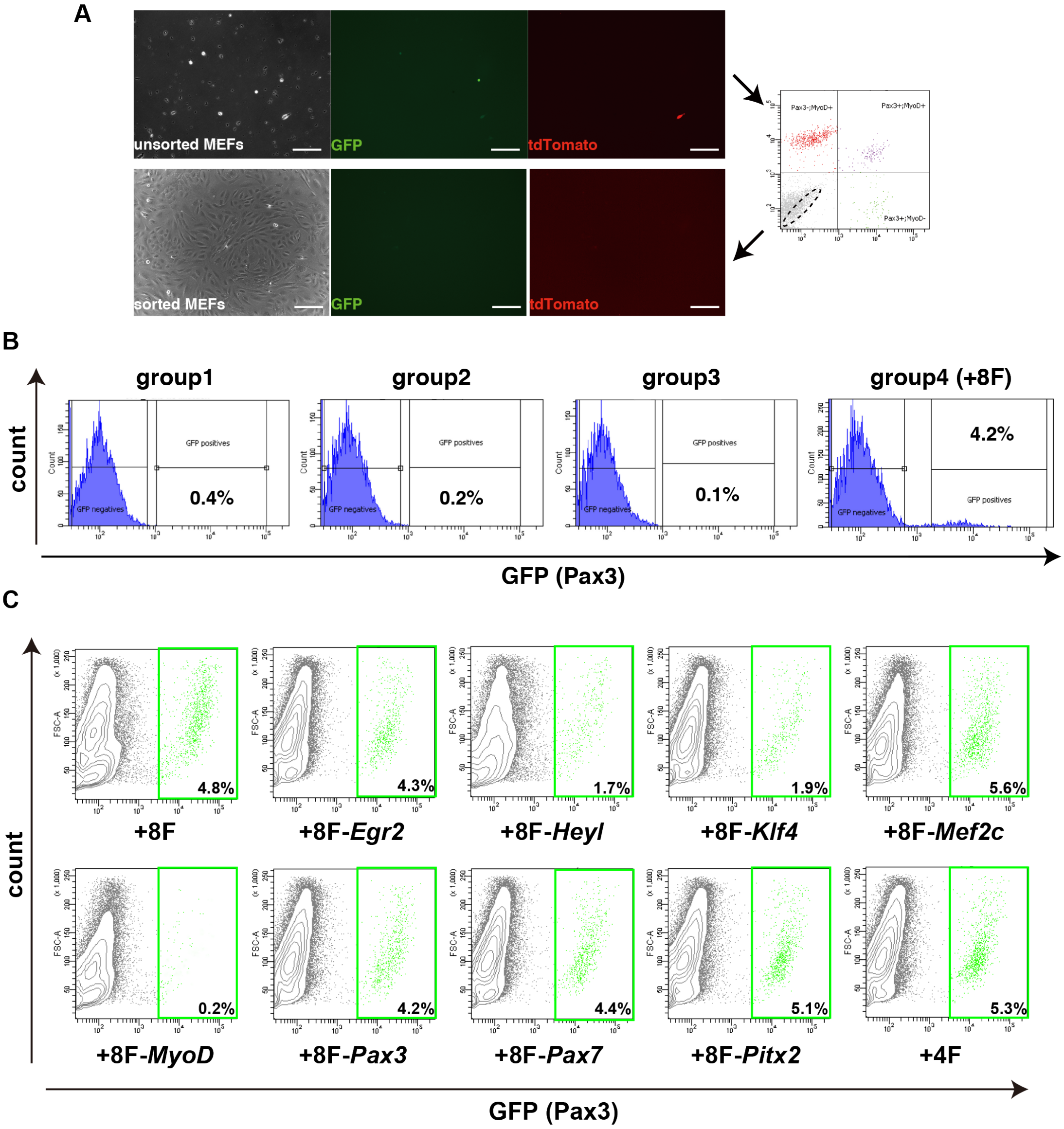

Figure S2. FACS analyses with Pax3$^{GFP/+}$ MEFs for reprogramming. (A) Fluorescent signals of GFP (Green) or tdTomato (Red) could be detected in mouse embryonic fibroblasts (MEFs) isolated from *Pax3-GFP; MyoD-tdTomato* embryos (upper panels). Sorted MEFs had no signal of any fluorescent proteins (lower panels). Scale bars; 50 µm. (B) FACS data of Pax3-GFP cells expressing selected candidates, named as Group1 (*Dmrt2, Foxp2, Hey1, Lbx1, Pbx1, Mef2d, Myf5, Six1, Tshz1, Tshz3*), Group2 (*Myog, Nfib, Pknox2, Prox1, Pere, Rora, Runx1, Sox6, Tbx1, Tbx15*), Group3 (*Tbx4, Tef, Tfdp2, Thra, Yy1, Zeb2, Zfp445, Zhx1, Zkscan17, Zkscan3*), Group4 (*Egr2, Heyl, Klf4, Mef2c, MyoD, Pax3, Pax7, Pitx2*) were used for infected into MEFs. (C) Narrowing down from 8 candidate transcription factors for PAX3-expressing myogenic cells derived from sorted MEFs.

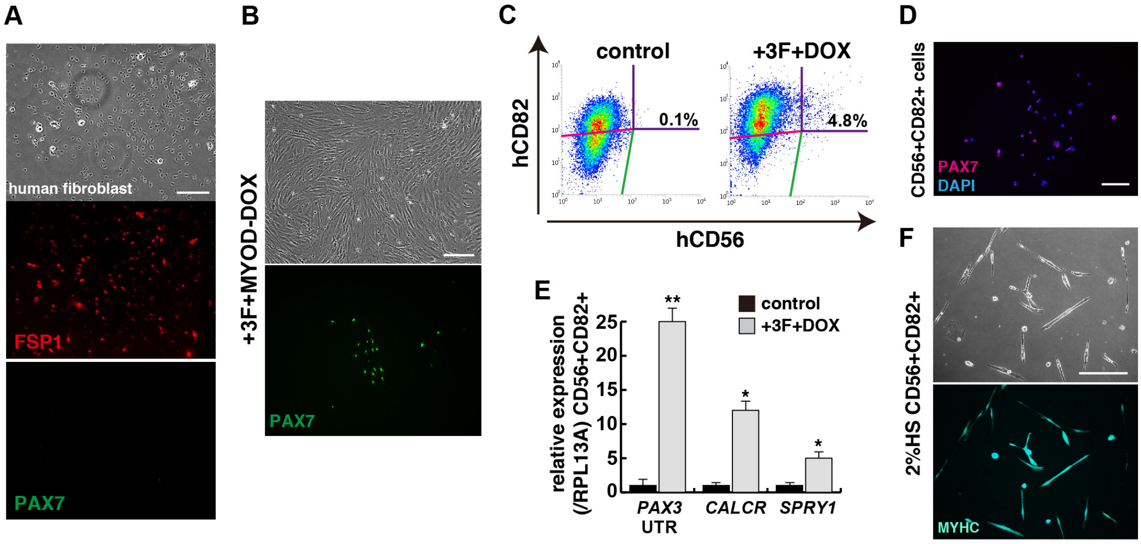

Figure S3. **4 transcription factors convert human fibroblasts into myogenic stem cells.** (A) Immunostaining of anti-FSP1 (labelled with Alexa594, red) and anti-PAX7 (labelled with Alexa488, green) antibodies with human dermal fibroblasts. (B) PAX3, HEYL, KLF4 (+3F) and transient DOX-treatment for 72 hours (+MYOD-DOX) induced PAX7-expressing cells from human dermal fibroblasts (labelled with Alexa488, green). (C) FACS profile of CD56 and CD82 cells from cultured human dermal fibroblasts with or without PAX3, HEYL, KLF4 and DOX treatment for 72 hours (control or +3F+DOX/control). (D) Immunostaining with anti-PAX7 antibody with CD56+CD82+ double positive sorted cells. PAX7; red, DAPI; blue. (E) Transcriptome analyses of induced CD56+CD82+ cells with or without the 4 factors. All error bars indicate ±SEM (n=3). *P*-values are determined by *t*-test from a two-tailed distribution. (F) MYHC (light blue) was stained with myogenic differentiated cells with 2% of horse serum (2%HS) from CD56+CD82+ sorted cells for 7 days. All scale bars; 50 μm.

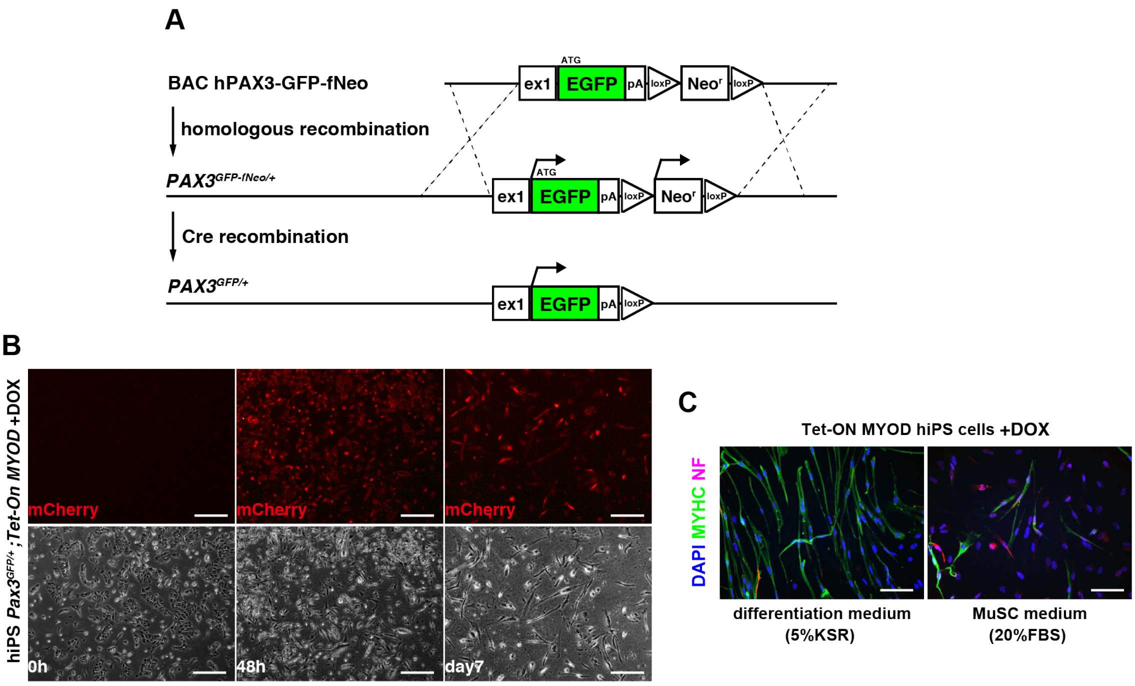

Figure S4. Strategy of GFP-targeting into the PAX3 knock-in and Tet-ON MYOD1 human iPS cells. (A) Schematic diagram of the human PAX3 locus and targeting construct. The BAC DNA construct contains more than 100kb of PAX3 genomic sequence. An EGFP reporter gene followed by floxed neomycin (fNeo) selection marker replaces the coding sequence in exon 1 of PAX3 (BAC *hPAX3-GFP-fNeo*). After homologous recombination in 201B7 hiPS cells, cells were selected by the administration of G418, and the knockin allele was confirmed by qPCR with genomic DNA and sequencing reaction ($PAX3^{GFP\text{-}fNeo/+}$). The fNeo cassette is removed with expression of CAG-Cre plasmid. This generates the $PAX3^{GFP/+}$ allele in hiPS cells. (B) $Pax3^{GFP/+}$*; Tet-On MYOD* hiPS cells were differentiated into myogenic cells with the administration of DOX for 7 days. (C) Immunostaining of terminally differentiated myotubes derived from hiPS cells with the administration of DOX for a week and 5% of Knockout Serum Replacement (KSR) was carried out according to an optimized protocol. (Tanaka et al. 2013, left panel). Neural cells appear in cultured cells with 20% of FBS instead of KSR (red, right panel). DAPI (blue), Myosin Heavy Chain (MyHC; labelled with Alexa488, Green), Neurofilament (NF; labelled with Alexa647, Red). All scale bar; 50 µm.

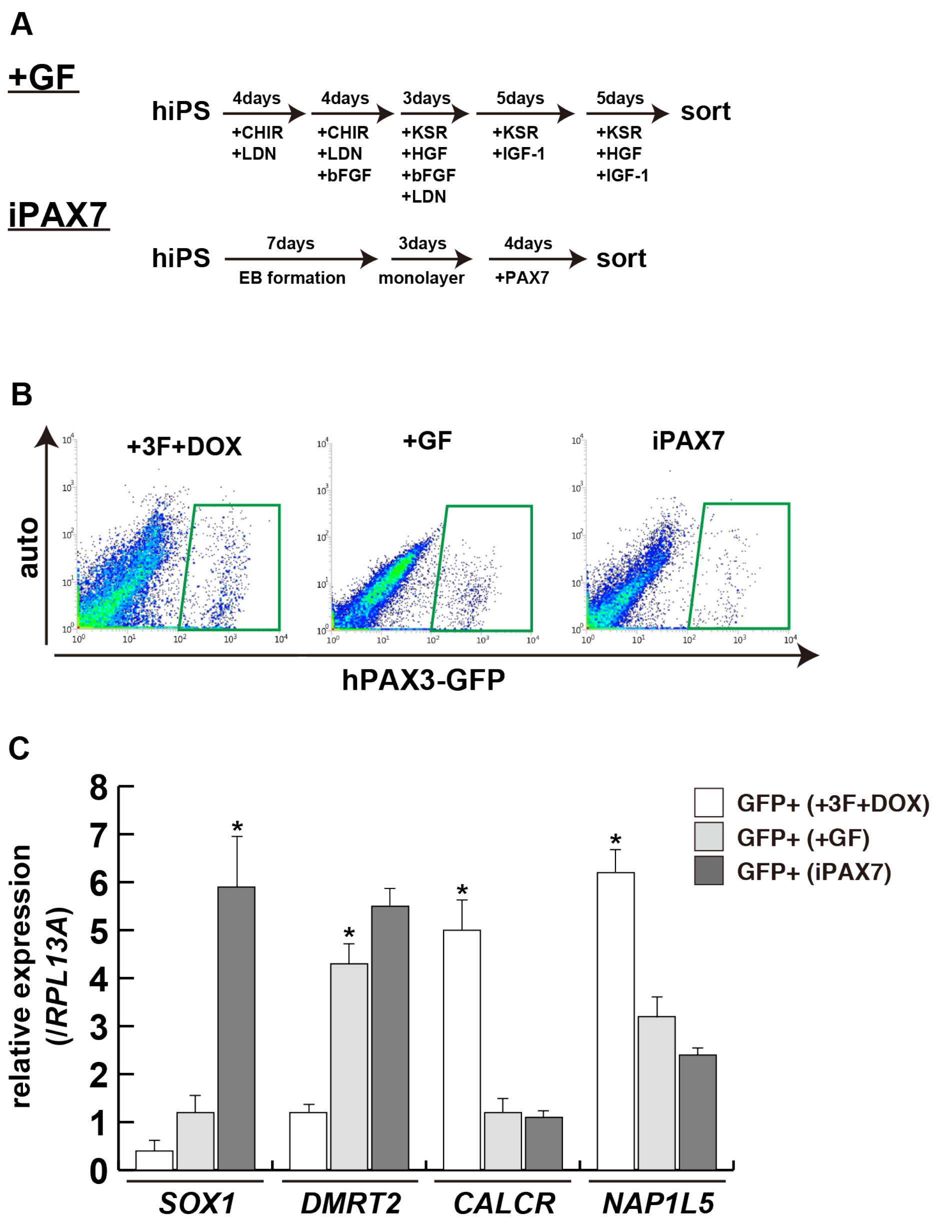

Figure S5. The comparison of induction methods for PAX3-expressing muscle stem cells from human iPS cells. (A) Reported schematic differentiation protocols for isolating PAX3-GFP myogenic cells derived from hiPS cells (iPAX7, Darabi et al. 2012.; +GF, Chal et al. 2015). (B) FACS profile of induced PAX3-GFP cells from hiPS cells. (C) Transcriptional analyses with PAX3-GFP positive cells induced by transient DOX treatment plus 3 transcription factors (+3F+DOX), +growth factors (+GF), and transient PAX7 (+iPAX7). Ectodermal and Mesodermal GFP-expressing cells, marked by *SOX1* (neural) and *DMRT2* (dermomyotome); *CALCL* and *NAP1L5* were used as markers for adult muscle satellite cells. All error bars indicate ±SEM (n=3). *P*-values are determined by Dunnett’s multiple-comparisons test. *P<0.01.

Table S2. **Summarized profile of Table S1**.

38 candidate genes which show high expression in Pax3-GFP muscle stem cells.

MuSC_E16	MuSC_1wk	MuSC_12wk	ESC	MEF	GSC	NSC	Gene Symbol
234.9	185.0	113.6	4.8	4.8	4.7	4.7	Dmrt2
2286.2	5709.4	13794.6	10.1	161.7	237.7	93.0	Egr2
263.4	278.7	278.0	8.4	118.4	7.7	13.7	Foxp2
466.4	674.9	892.0	100.2	23.8	252.3	78.9	Hey1
189.4	790.0	749.6	7.5	7.3	7.4	7.2	Heyl
536.2	415.1	1637.4	224.4	262.6	93.6	10.4	Klf4
140.2	86.1	74.6	5.0	5.1	4.9	4.9	Lbx1
765.7	420.5	509.4	9.5	178.2	8.5	184.5	Pbx1
326.3	725.9	1909.8	6.3	22.7	8.8	188.5	Mef2c
385.8	521.1	381.8	16.4	109.3	76.7	149.0	Mef2d
5075.5	4503.2	9603.6	10.1	10.2	8.6	8.8	Myf5
960.1	2266.2	3791.0	3.5	3.5	3.4	3.4	Myod1
10523.2	12490.2	4666.3	6.1	6.1	5.8	5.8	Myog
6651.9	7772.7	5271.2	23.5	425.0	77.3	2492.1	Nfib
231.6	1174.9	1542.6	17.9	20.7	15.9	171.2	Pax3
3043.4	3078.9	4765.1	4.6	4.6	4.5	4.4	Pax7
1936.7	2850.5	1779.0	92.1	164.1	9.9	9.6	Pitx2
321.8	758.6	224.2	6.6	9.2	5.4	43.2	Pknox2
161.6	733.3	473.6	7.6	7.0	6.7	14.0	Prox1
804.1	1129.9	864.9	162.5	144.2	367.6	392.3	Rere
319.0	543.5	2214.3	41.7	24.8	45.9	41.8	Rora
125.3	217.6	1614.6	6.2	28.0	8.7	5.6	Runx1
429.6	399.2	301.6	27.3	80.0	220.9	11.3	Six1
406.8	481.9	281.1	14.4	17.6	13.5	153.4	Sox6
182.4	228.1	104.4	5.9	7.9	7.1	5.9	Tbx1
306.4	302.1	258.5	6.6	248.6	9.2	64.5	Tbx15
76.0	70.6	127.8	5.5	16.5	5.2	5.3	Tbx4
816.2	850.9	657.6	194.7	229.1	438.6	315.9	Tef
2527.8	2323.1	1054.0	610.8	128.8	198.6	265.8	Tfdp2
242.2	702.1	447.1	5.8	34.3	16.7	222.7	Thra
2499.5	2727.7	1730.1	7.1	452.2	15.8	466.1	Tshz1
686.8	740.7	934.0	16.5	129.1	16.6	86.2	Tshz3
1336.7	995.4	1132.6	352.3	472.0	61.7	594.6	Yy1
854.9	4935.4	3900.5	11.5	249.2	8.0	147.3	Zeb2
441.2	887.0	525.6	165.1	141.6	177.6	273.0	Zfp445
596.6	1129.1	743.8	145.4	270.0	128.9	364.6	Zhx1
679.4	871.6	733.0	374.6	156.2	49.4	463.5	Zkscan17
1039.6	755.8	678.5	77.9	164.8	157.7	308.7	Zkscan3

Table S3. Primers for the expression analysis by RT-qPCR of the mRNAs indicated.

for mouse

Gene	Sequence	Target position	Product
Rpl13a	5'-GTGGTCCCTGCTGCTCTCAAG-3'	exon6	151bp
	5'-CGATAGTGCATCTTGGCCTTTT-3'	exon7	
Pax7 (UTR)	5'-CCCTTCCTACAGGAAACCCTCT-3'	exon10	72bp
	5'-CTGAACCAGACCTGGACGCG-3'	exon10	
MyoD (UTR)	5'-GCGCTCTTCCTTTCCTCATAG-3'	exon2	90bp
	5'-GGGCTCCAGAAAGTGACAAAC-3'	exon3	
Myog	5'-CAACCAGGAGGAGCGCGATCTCCG-3'	exon1	93bp
	5'-GGCGCTGTGGGAGTTGCATTCACT-3'	exon2	
Calcr	5'-ATCTGGTGCGGCGGGAT-3'	exon10	94bp
	5'-CCCTCGCAGAGCATCCAGAA-3'	exon12	
Spry1	5'-ATGGATTCCCCAAGTCAGCAT-3'	exon3	93bp
	5'-CCTGTCATAGTCTAACCTCTGCC-3'	exon3	
Sdc4	5'-GTCCCCGGAGAGTCGATTC-3'	exon1	193bp
	5'-GCACCAAGGGCTCAATCACTT-3'	exon3	
Myh3	5'-TCCAAACCGTCTCTGCACTGTT-3'	exon17	84bp
	5'-AGCGTACAAAGTGTGGGTGTGT-3'	exon18	
Myh7	5'-ATGCTGACAGATCGGGAGAA -3'	exon	181bp
	5'-GGTTGGCTTGGATGATTTGA-3'	exon	
Myh1	5'-TCTGCAGACGGAGTCAGGT-3'	exon33	94bp
	5'-TTGAGTGAATGCCTGTTTGC-3'	exon34	
Sox1	5'-CCTCGGATCTCTGGTCAAGT-3'	exon1	107bp
	5'-GCAGGTACATGCTGATCATCTC-3'	exon1	
Sox10	5'-ATGTCAGATGGGAACCCAGA-3'	exon2	74bp
	5'-GTCTTTGGGGTGGTTGGAG-3'	exon3	

for human

RPL13A	5'-CCCTGGAGGAGAAGAGGAAA-3'	exon11	91bp
	5'-ACGTTCTTCTCGGCCTGTTT-3'	exon12	
PAX3	5'-AGGAAGGAGGCAGAGGAAAG-3'	exon6	174bp
	5'-CAGCTGTTCTGCTGTGAAGG-3'	exon10	
PAX3(UTR)	5'-AAGCCAGCTGACTGTTCCAG-3'	exon12	74bp
	5'-CCGCAAGATGTTGTTGACAT-3'	exon12	
PAX7	5'-GGGATTCCCTTTGGAAGTGT-3'	exon1-2	198bp
	5'-CGGCAAAGAATCTTGGAGAC-3'	exon2	
CALCR	5'-CCCTTTGCTTCTATTGAGCTG-3'	exon2	69bp
	5'-GGTAATAGCATGGATAGTGGTTGGt-3'	exon3	
MYF5	5'-CTATAGCCTGCCGGGACAGA-3'	exon1	95bp
	5'-TGGACCAGACAGGACTGTTACAT-3'	exon3	
DMRT2	5'-GAACCACCAAGCAAGGACTTC-3'	exon5	75bp
	5'-CCCAGACCCTGAATACTGCAT-3'	exon5	
NFIX	5'-TGACTCCTCCATCACCTTCA-3'	exon8	71bp
	5'-GGGTCCGATGCTGACAAA-3'	exon9	
NAP1L5	5'-GTGTGCATGGACCTTGGAG-3'	exon1	62bp
	5'-CCTCCTCGTCATCCTCGTACT-3'	exon1	
SPRY1	5'-TCCCTGGTCATAGGTCTGAAAG-3'	exon4	187bp
	5'-TGCCGGTTACAGGCCAAAC-3'	exon4	
MSGN1	5'-GGAGAAGCTCAGGATGAGGA-3'	exon1	145bp
	5'-GTCTGTGAGTTCCCCGATGT-3'	exon1	
TBX6	5'-GAACGGCAGAAACTGTAAGAGG-3'	exon5	101bp
	5'-GTGTGTCTCCGCTCCCATAG-3'	exon6	
PDGFRa	5'-AACCGTGTATAAGTCAGGGGA-3'	exon4	126bp
	5'-ATTTCTTCCAGCATTGTGAT-3'	exon6	
SOX1	5'-ACCAGGCCATGGATGAAG-3'	exon1	67bp
	5'-CTTAATTGCTGGGGAATTGG-3'	exon1	
SOX10	5'-CGGACCAGTACCCGCACCT-3'	exon2	87bp
	5'-GGCGCTTGTCACTTTCGTTCA-3'	exon3	
